# A broadly protective vaccine against cutaneous human papillomaviruses

**DOI:** 10.1038/s41541-022-00539-0

**Published:** 2022-10-10

**Authors:** Filipe Colaco Mariz, Kathrin Balz, Manuela Dittrich, Yueru Zhang, Fan Yang, Xueer Zhao, Angelo Bolchi, Simone Ottonello, Martin Müller

**Affiliations:** 1 German Cancer Research Center, Im Neuenheimer Feld 242, 69120 Heidelberg, Germany; 2 Department of Chemistry, Life Sciences and Environmental Sustainability, University of Parma, 43124 Parma, Italy

**Keywords:** Protein vaccines, Viral infection

## Abstract

Skin colonization by human papillomavirus (HPV) is typically related to inconspicuous cutaneous infections without major disease or complications in immunocompetent individuals. However, in immunosuppressed patients, especially organ transplanted recipients, cutaneous HPV infections may cause massive, highly spreading and recurrent skin lesions upon synergism with UV-exposure. Current HPV prophylactic vaccines are not effective against cutaneous HPV types (cHPV). By applying a modular polytope-based approach, in this work, we explored different vaccine candidates based on selected, tandemly arranged cHPV-L2 epitopes fused to thioredoxin (Trx) as a scaffold protein. Upon conversion to heptameric nanoparticles with the use of a genetically fused oligomerization domain, our candidate Trx-L2 vaccines induce broadly neutralizing immune responses against 19 cHPV in guinea pigs. Similar findings were obtained in mice, where protection against virus challenge was also achieved via passive transfer of immune sera. Remarkably, immunization with the candidate cHPV vaccines also induced immune responses against several mucosal low- and high-risk HPV types, including HPV16 and 18. Based on cumulative immunogenicity data but also on ease and yield of production, we identified a lead vaccine candidate bearing 12 different cHPV-L2 epitopes that holds great promise as a scalable and GMP production-compatible lead molecule for the prevention of post-transplantation skin lesions caused by cHPV infection.

## Introduction

The carcinogenic role of some human papillomavirus types (HPV) has been firmly established^1^. About 5% of all cancers globally result from HPV infection^2^, with estimates that 604,000 women^3^ and 60,000 men^2^ develop HPV-related cancers annually. Over 200 different HPV types are currently described with varying tropism for mucosa or skin^4^. The skin of all individuals constantly harbors a plethora of HPV, which are rarely associated to malignant pathologies. A number of HPV phylogenetically classified into the beta-genus (beta-HPV) have been assigned as commensals, because of their ubiquitous presence (>90%) in skin swabs or hair bulbs of healthy individuals with no visible lesions or warts^5–8^. In fact, the vast majority (>95%) of all viral DNA sequences found in skin samples correspond to papillomaviruses, mostly from the beta- and the gamma-genera^9^. The controlled adaptation to commensal cutaneous HPV can be, however, de-regulated under immune suppression conditions, as commonly observed in solid organ transplant recipients (OTR)^10,11^.

The occurrence of cutaneous warts in OTR has been shown to tightly correlate with the duration of iatrogenic immune suppression^10^. Over 90% of the OTR are reported to acquire cutaneous warts within five years post-transplantation^11^. Although the mortality associated to these post-transplantation skin lesions is low^12^, the ablative therapies needed for treatment are painful, disfiguring and may not provide a long-lasting cure given the recurrent characteristics of these skin lesions in OTRs^13^.

In addition to a considerable patient morbidity, the profuse and recurrent cutaneous warts observed in OTR are frequently accompanied by keratinocyte-associated malignant lesions known as non-melanoma skin cancer (NMSC)^14,15^. A subgroup of NMSC classified as squamous cell carcinoma (SCC), represents the most common malignancy in this patients’ cohort, with a 60- to 250-fold increased risk of incidence compared to the general population^10,16–18^. In OTR, SCC represents the most aggressive outcome with a considerably higher risk of metastasis and death compared to the general population^19^. These clinical observations were, in fact, preceded by the earlier discovery of beta-HPV (e.g. HPV types 5, 8, 9, 15, 36, 38, 76, 92, and 96) in SCC from patients affected by a rare genetic skin disease known as *epidermodysplasia verruciformis* (EV), which is characterized by increased susceptibility to widespread infection by beta-HPV, mainly on sun-exposed sites. A close clinical and morphological resemblance between the SCC observed in OTR and the HPV-induced warts present in EV-patients has also been documented^20^. Non-classical, acquired forms of EV have been also documented in HIV/AIDS patients^21^ and in other inherited immune dysfunctions^22^. Nevertheless, beta-HPV were also detected in NMSC of non-EV patients, with a higher incidence in transplanted patients compared to the general population^23,24^. Serological studies substantiated these findings by correlating the development of cutaneous squamous cell carcinoma in OTR to the presence of antibodies against not only beta-^25,26^ but also gamma-HPV types (e.g. HPV types 4, 50, 88, and 95)^27^. Alpha-HPV types known to colonize the skin (e.g. HPV types 2, 3, and 10) have also been associated to cases of widespread, potentially disfiguring cutaneous HPV infections, a condition known as generalized verrucosis commonly observed in patients with underlying immunodeficiency status (acquired or congenital)^28,29^. In our opinion, this scenario illustrates the need to develop a broadly immunogenic vaccine, rather than focusing on a subset of types clustered in a single papillomavirus genus.

According to the global database on donation and transplantation, over 150,000 solid organs were transplanted globally in 2019, which represents a 5% increase compared to the previous year^30^. A solid organ transplant in the United States may range from USD 442,000 for a single kidney transplant to over 2.6 million for combined heart and lung transplantation^31^. In Germany, around 3000 people are subjected to chronic immune suppression every year due to organ transplantation^32^, while the long-term oral maintenance immunosuppression has been estimated to cost USD 2500 monthly per patient^33^. These expenditures are augmented by the frequent development of skin lesions in OTRs. Policy makers in the United States and United Kingdom recommend that OTRs should be seen in dedicated surveillance dermatology clinics, given the high association of HPV-related warts and NMSC in these patient cohort. About 80% and 54% of transplanted patients in Australia^34^ and the United Kingdom^35^ are diagnosed with NMSC within 20 years of immunosuppression following transplantation. In the United States, the annual expenses for NMSC-related medical care are estimated to cost USD 650 million^36^.

Currently available HPV vaccines based on virus-like particles formed by the major capsid protein L1 are highly safe and effective, yet restricted to a type-specific prevention only acting against low- and high-risk mucosal HPV types associated to anogenital cancer. In recent years, we have documented the immune reactivity and vaccine translational potential of the minor capsid protein L2^37^, which due to its higher sequence conservation can afford a much broader (cross-)protection against multiple HPV types. This paved the way to the development of PANHPVAX^38^, an L2-based vaccine candidate currently undergoing a first-in-human, single center, open-label, phase I, dose escalation trial in healthy volunteers (NCT05208710). The antigen will be formulated with cyclic-di-AMP as adjuvant. Following the same modular polytope-based approach applied to PANHPVAX, we generated a number of antigens displaying several conserved cHPV L2 epitopes grafted to the display site of a thermally stable thioredoxin (Trx) scaffold protein from the archaeon *Pyrococcus furiosus*, which was then further assembled into more immunogenic heptameric nanoparticle structures. Herein, we systematically investigated the immunogenicity of a series of candidate polytopic vaccines targeting a panel of cutaneous HPV types clinically associated to post-transplantation cutaneous lesions. We thus identified a leading vaccine candidate bearing 12 different cHPV-L2 epitopes that induce neutralizing antibodies against 19 cHPV in guinea pigs, affords protective immunity in a murine challenge model of HPV infection, and is amenable to a GMP-compatible production.

## Results

### OVX313-mediated oligomerization of polytopic PfTrx-L2 cHPV antigens enhances immunogenicity in mice

We have previously demonstrated that the PANHPVAX vaccine, which specifically targets low- and high-risk mucosal but not cutaneous HPV, also induces neutralizing antibodies against some cHPV types^38^. Nevertheless, we reasoned that due to the great heterogeneity and phylogenetic diversity of cutaneous HPV the protection afforded by PANHPVAX may not be sufficiently comprehensive (Supplementary Fig. 1). In order to achieve protection specifically directed against cutaneous HPV, we thus designed two PfTrx-L2-based polytopic antigens comprising the L2 (aa 20–38) cross-neutralization epitopes of six and nine cutaneous HPV, designated as PfTrx-L2c6mer and PfTrx-L2c9mer, respectively. The L2 epitope of the cHPV was selected to correspond to the region aa20–38 of HPV16 L2, and with the six and nine polytopic configurations, at least one cHPV of alpha-, beta-, gamma- and mu-papillomavirus genera is represented. Given the enhanced anti-mucosal HPV immunogenicity previously found to be conferred by genetic fusion of the basic PfTrx-L2 antigens^38–40^ to the OVX313 heptamerization domain, we also investigated the effect of structured oligomerization on the immunogenicity of the PfTrx-L2c6mer and PfTrx-L2c9mer antigens.

**Fig. 1:**
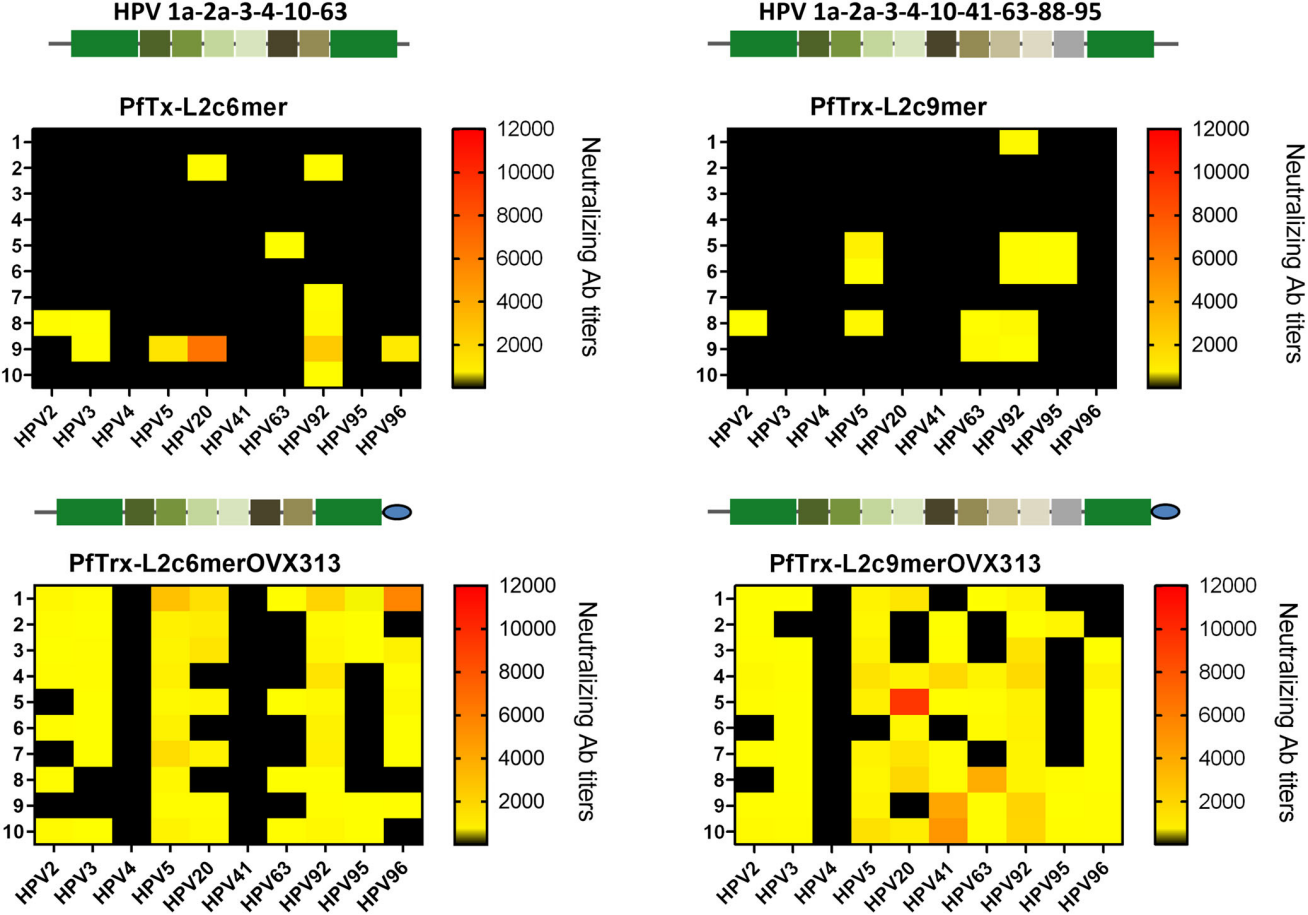
OVX313-mediated heptamerization boosts the immunogenicity of both the PfTrx-L2c6mer and the PfTrx-L2c9mer antigens in mice. Heat maps show the neutralizing antibody titers against 10 cutaneous HPV types, measured by the L1-PBNA in sera from 10 mice (each row represents one serum) immunized with 20 µg of the four cHPV PfTrx-L2 antigens. Antibody titers ranging from 50 to 12,000 are color-coded in yellow to red; black color indicates EC50 values <50 (no neutralization). At the top of each heat map, the antigen schematics illustrate the HPV L2 epitopes (squares) genetically inserted into the PfTrx protein (rectangle), and the presence or absence of the OVX313 domain (blue circle) C-terminally.

We found that similarly to PANHPVAX, OVX313-mediated heptamerization conferred a strikingly increased immunogenicity to the cutaneous L2 polytopes, compared to the corresponding OVX313-lacking antigens (Fig. 1). In fact, an overall very weak immune response, with low antibody titers and a very modest percentage (%) of responders to cutaneous HPV types (50% of mice responded to HPV92 only; see Fig. 1 and Supplementary Tables 1 and 2), was observed upon vaccination with the monomeric form of the antigens. Conversely, 2- to 7-fold higher mean neutralizing antibody titers against HPV2, HPV3, HPV5, HPV20, HPV92, and HPV96 were detected in 70–100% of the mice immunized with the oligomeric form of the PfTrx-L2c6merOVX313 antigen (Fig. 1 and Supplementary Table 2). An even stronger response was observed with the three further cHPV L2 epitopes (HPV41, HPV88, and HPV95) added to the expanded PfTrx-L2c9merOVX313 antigen. This antigen induced up to 30-fold higher mean neutralizing antibody titers compared to its monomeric counterpart. Interestingly, the PfTrx-L2c9merOVX313 antigen-induced neutralizing antibodies against HPV41 in 80% of the mice (as opposed to a complete lack of response in the case of PfTrx-L2c6merOVX313), suggesting that the presence of the HPV41 L2 epitope is required to trigger strongly neutralizing antibody responses against this particular HPV type. However, a very weak immune response to HPV4 and HPV95 was observed even when the corresponding epitopes were incorporated into both forms of the 9mer antigen.

**Fig. 2:**
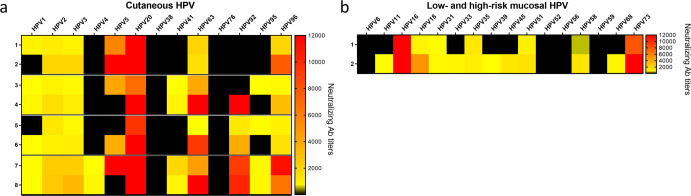
PfTrx-L2c6mer and PfTrx-L2c9mer polytopes induce a broad immune response in guinea pigs. Heat maps show the neutralizing antibody titers to cutaneous HPV types (**a**) measured by the L1-PBNA in sera from eight guinea pigs immunized with 30 µg of the PfTrx-L2c6mer (rows 1 and 2), PfTrx-L2c9mer (rows 3 and 4), PfTrx-L2c6merOVX313 (rows 5 and 6) and PfTrx-L2c9merOVX313 (rows 7 and 8) antigens. **b** The immunogenicity of 30 µg of PfTrx-L2c6mer (row 1) and PfTrx-L2c9mer (row 2) against low- and high-risk mucosal HPV types was also assessed by the L1-PBNA. Antibody titers ranging from 50 to 12,000 are color-coded in yellow to red; black color indicates EC50 values <50 (no neutralization).

A rather different situation was observed in guinea pigs, where the monomeric PfTrx-L2c6mer and PfTrx-L2c9mer antigens displayed a strong immunogenicity against a broad range of cutaneous and mucosal HPV types (Fig. 2a, b), with generally higher neutralizing antibody titers compared to those measured in mice. Still, a better immune performance of the oligomerized PfTrx-L2c9merOVX313 antigen was also apparent in guinea pigs (Fig. 2a). In particular, we found increased mean neutralizing antibody titers against HPV2, HPV3, HPV41, HPV92, and HPV96 in the sera of guinea pigs immunized with PfTrx-L2c9merOVX313 compared to its monomeric version (Supplementary Table 3). Moreover, unlike the mice, the guinea pigs responded against HPV4 and HPV95. Anti-HPV1 and HPV5 mean neutralizing antibody titers were nearly the same in animals immunized with either PfTrx-L2c6merOVX313 or PfTrx-L2c9merOVX313, and neither of the antigens induced a neutralizing response against HPV38 and HPV76 (Supplementary Table 3).

### A polytopic antigen specifically designed for cutaneous HPV is necessary to induce a strong and comprehensive cHPV-specific immune response

In a previous study^38^, we demonstrated that PANHPVAX, a non-cHPV dedicated vaccine prototype similarly built on a PfTrx-OVX313 scaffold, is able to induce cross-neutralizing antibody responses against some cutaneous HPV. Although promising, these observations were derived from a limited number of immunized guinea pigs, which are outbred animals with a known ability to mount a particularly strong immune response to our PfTrx-L2 antigens compared to inbred Balb/c mice. Therefore, we performed a more comprehensive, side-by-side comparison of PANHPVAX, PfTrx-L2c6merOVX313, and PfTrx-L2c9merOVX313 immunogenicity in mice.

**Fig. 3:**
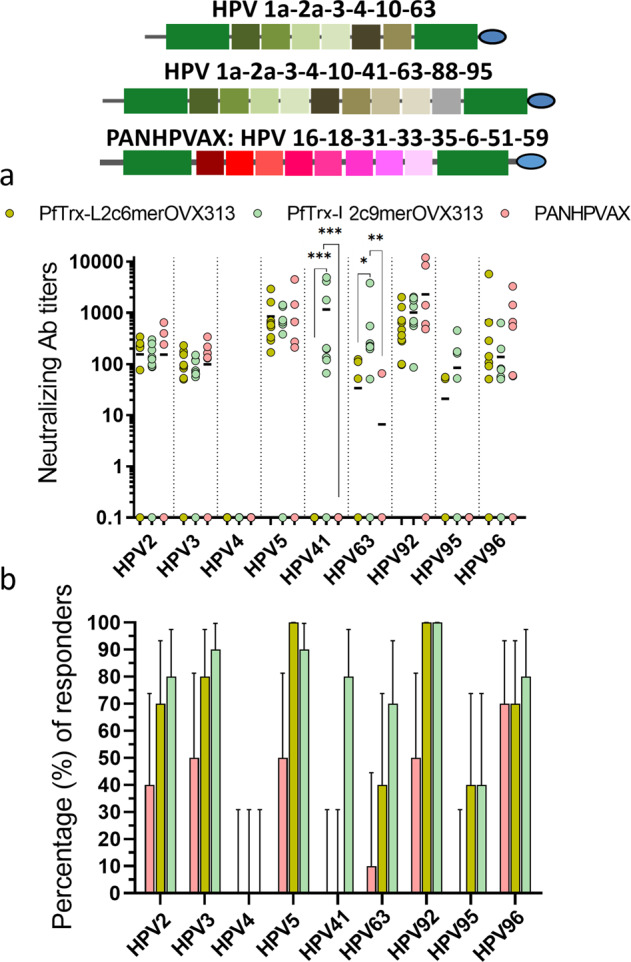
PfTrx-L2c6merOVX313 and PfTrx-L2c9merOVX313 outperform the PANHPVAX vaccine with regard to broadness of the induced immune response to cutaneous HPV types. **a** Neutralizing antibody titers to nine HPV types measured by the L1-PBNA in sera from 10 mice immunized with 20 µg of the PfTrx-L2c6merOVX313 antigen, PfTrx-L2c9merOVX313 and PANHPVAX (note the antigen/color designation on top of the graph). Each dot represents the EC50 value derived from one animal serum; horizontal lines correspond to the mean of neutralizing antibody titers. The three antigens are illustrated on the top of panel a. A *P-*value of ≤0.05 was considered significant. *P < 0.05; **P < 0.01; ***P < 0.001, as determined by the nonparametric Mann–Whitney test. **b** Percentage (%) of responders showing neutralizing antibody levels induced by the three antigens. The lines on top of each bar represent the upper 95% confidence interval. EC50 values <50 were considered non-neutralizing and therefore set to 0.1.

The three antigens induced comparable neutralizing antibody responses to the cutaneous HPV types 2, 3, 5, 92, and 96 (Fig. 3 and Supplementary Table 4). However, except for the more consistent response against HPV96, PANHPVAX induced cross-neutralizing antibodies in no more than 50% of the immunized mice, whereas a generally higher response rate was achieved with PfTrx-L2c6merOVX313 and PfTrx-L2c9merOVX313 (Fig. 3b and Supplementary Table 5). In particular, and in keeping with our previous findings, only weak neutralizing responses against the non-vaccine types HPV41, HPV63, and HPV95 were detected in mice immunized with PANHPVAX. Altogether, these data suggest that the incorporation of specific cutaneous HPV L2 aa 20-38 epitopes is required for inducing a robust immune response capable of neutralizing an extended range of cutaneous HPV types.

### Higher-complexity polytopic candidate vaccines induce neutralizing antibodies against 19 cutaneous HPV types

**Fig. 4:**
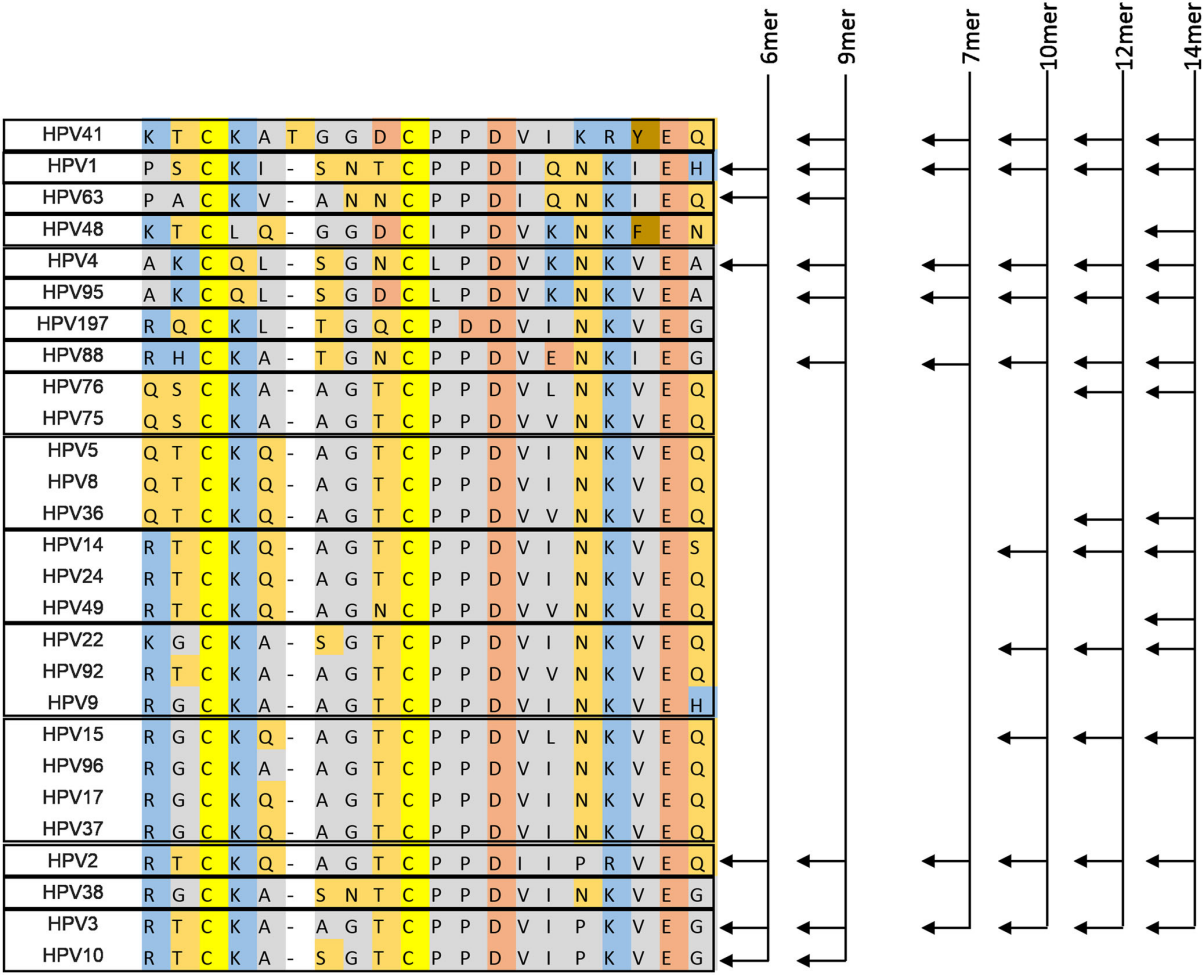
Multiple alignment of 27 cutaneous HPV L2 aa 20-38 epitopes. At the left side of the scheme, the amino acid sequence similarities are shown by different colors, and epitopes with a similatiry >95% are grouped by boxes. On the right, the presence of each corresponding L2 aa 20–38 epitope in each of the designed polytopic antigens is highlighted by arrows.

Prompted by the superior performance of PfTrx-L2c9merOVX313 compared to its 6mer counterpart, we set out to further modulate, and ultimately diversify the vaccine-incorporated cHPV L2 epitopes. This led us to design four novel candidate vaccines -one bearing a shortened polytope (PfTrx-L2c7merOVX313) and three (PfTrx-L2c10merOVX313, PfTrx-L2c12merOVX313, and PfTrx-L2c14merOVX313) with further expanded polytopes (Fig. 4)—whose immunogenicity was assessed in mice and guinea pigs, and compared with that of PfTrx-L2c9merOVX313.

**Fig. 5:**
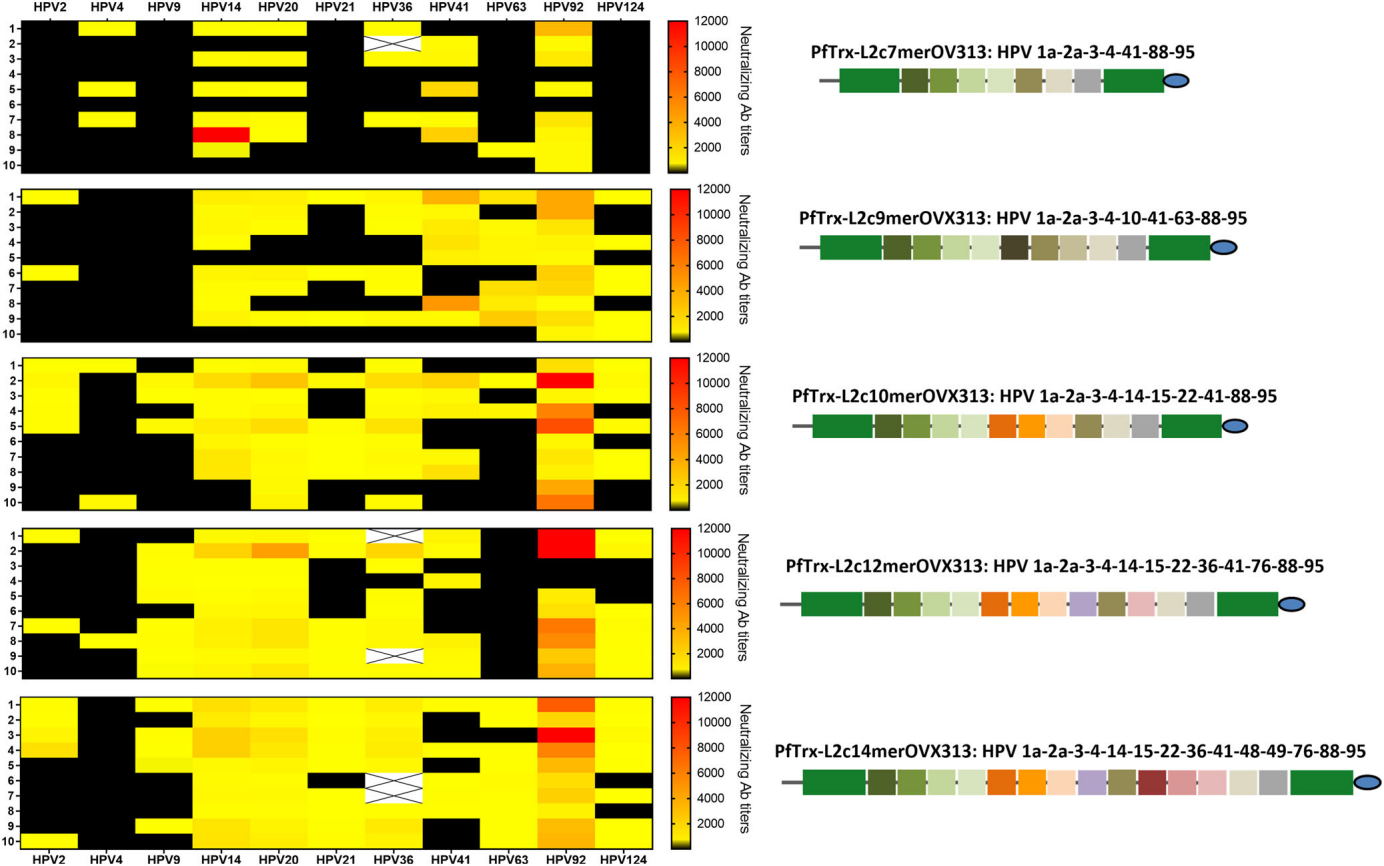
Two high-complexity candidate vaccines (12mer and 14mer) induce broadly neutralizing antibody responses to cutaneous HPV types. Heat maps show neutralizing antibody titers to 11 cHPV types measured by the L1-PBNA in sera from 10 mice immunized with 20 µg of the indicated antigens (each row represents one serum). Antibody titers ranging from 50 to 12,000 are color-coded in yellow to red; the black color indicates EC50 values <50 (no neutralization). A cross mark indicates sera that were not tested for HPV36 due to a limiting sample volume.

**Fig. 6:**
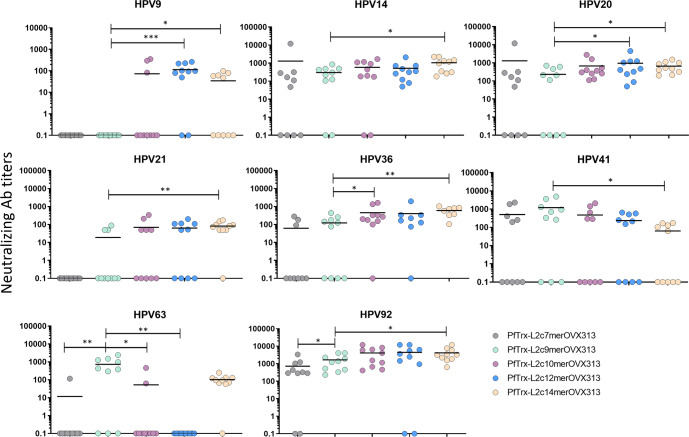
The PfTrx-L2c12merOVX313 and PfTrx-L2c14merOVX313 candidate vaccines induce enhanced immune responses to cutaneous HPV types compared to PfTrx-L2c9merOVX313. Neutralizing antibody titers to eight HPV types measured by the L1-PBNA in sera from mice immunized with 20 µg of the indicated Trx-L2 antigens. Each dot represents the EC50 value measured in one animal serum; horizontal lines (per column) correspond to the mean antibody titers. A *P*-value of ≤0.05 was considered significant. *P < 0.05; **P < 0.01; ***P < 0.001, as determined by the nonparametric Mann–Whitney test. EC50 values <50 were considered non-neutralizing and therefore set to 0.1.

The heat maps in Fig. 5 show the neutralizing antibody titers of mouse sera for 11 cutaneous HPV types and highlight an overall tendency toward an increasingly broad immune response with the introduction of more diverse and structurally more complex polytopes. Compared to PfTrx-L2c9merOVX313, we found increased titers of neutralizing antibodies against HPV9, HPV14, HPV20, HPV21, HPV36, HPV92 and HPV124, induced by the PfTrx-L2c12merOVX313 and the PfTrx-L2c14merOVX313 candidate vaccines (Figs. 5 and 6, Supplementary Table 6). The sole exception was HPV41, against which, higher neutralizing antibody titers were induced by the PfTrx-L2c9merOVX313 antigen (Figs. 5 and 6, Supplementary Table 6). While essentially similar responses to HPV63 were induced by the PfTrx-L2c9merOVX313 and the PfTrx-L2c14merOVX313 antigens, significantly lower neutralizing antibody titers were found upon immunization with the PfTrx-L2c7merOVX313, PfTrx-L2c10merOVX313 and PfTrx-L2c12merOVX313 candidate vaccines (Figs. 5 and 6, Supplementary Table 6). We have presently no explanation for these observations, considering that the HPV41 epitope was kept in all the antigens, and the HPV63 epitope is absent in the PfTrx-L2c14merOVX313. The unique presence of amino acids Ala (A) and Asn (N) between the cysteine residues of HPV49 L2 epitope, which are also conserved in the HPV63 L2 epitope (Fig. 4), may trigger the production of cross-neutralizing antibodies against the latter one. Interestingly, a slightly better anti-HPV4 response was elicited by the PfTrx-L2c7merOVX313 antigen, which corresponds to the least complex of all (Fig. 5).

**Fig. 7:**
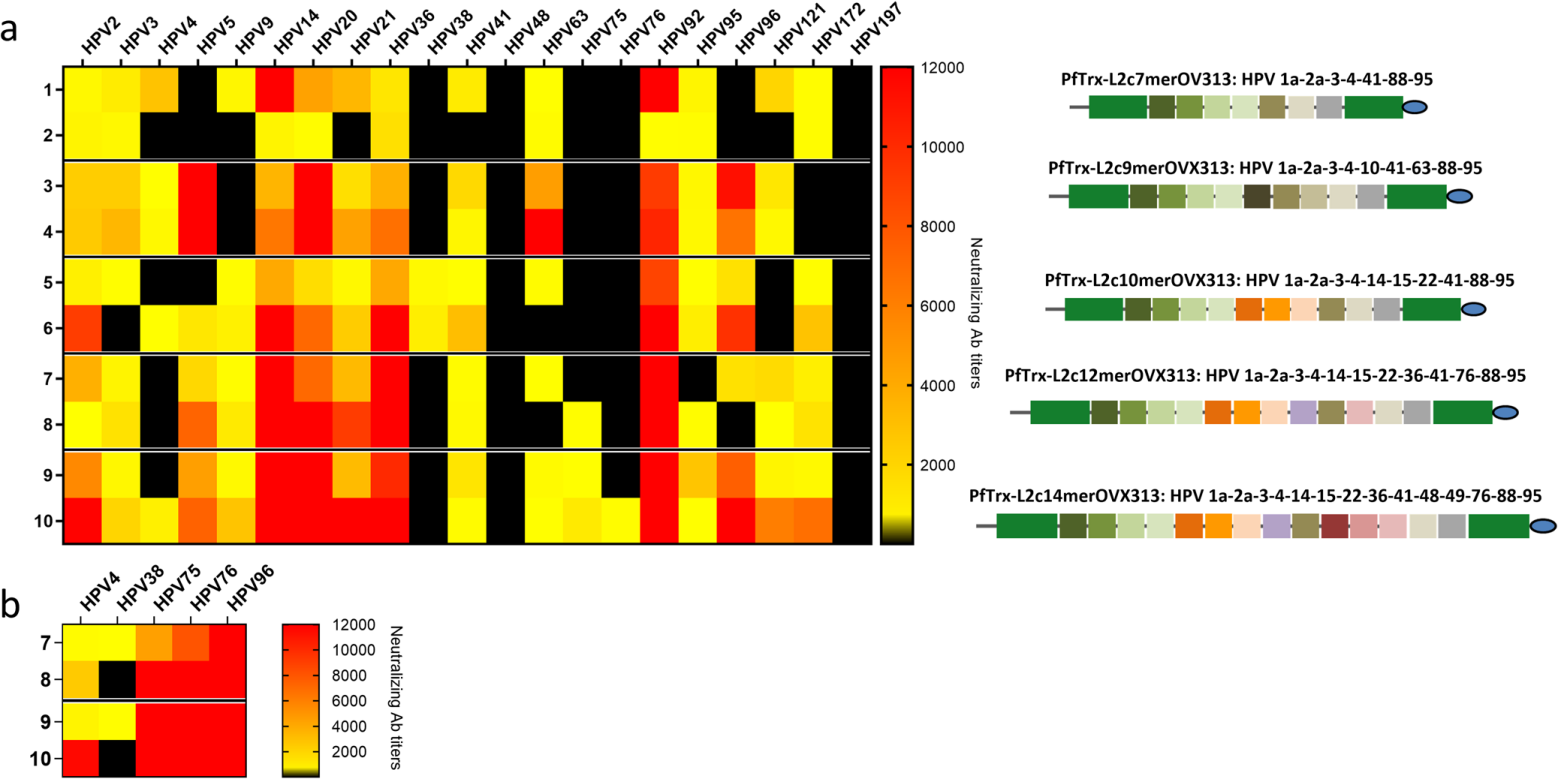
PfTrx-L2c12merOVX313 and PfTrx-L2c14merOVX313 antigens induced neutralizing antibodies against 19 cutaneous HPV types in guinea pigs. **a** Neutralizing antibody titers to the 21 indicated HPV types measured by the L1-PBNA in sera from two guinea pigs immunized with 30 µg of the five indicated PfTrx-L2-OVX313 antigens. Antibody titers ranging from 50 to 12,000 are color-coded in yellow to red; black color indicates EC50 values <50 (no neutralization). **b** Neutralizing antibody titers to five HPV types (indicated on the top) measured by the FC-PBNA in sera from two guinea pigs immunized with 30 µg of the PfTrx-L2c12merOVX313 and the PfTrx-L2c14merOVX313 antigens.

The above results were confirmed and extended by a more comprehensive analysis performed in guinea pigs, which revealed an immune response against 16 and 18 (out of a total of 21) cutaneous HPV types induced by the PfTrx-L2c12merOVX313 and PfTrx-L2c14merOVX313 candidate vaccines, respectively (Fig. 7a). Importantly, the use of the more sensitive FC-PBNA revealed the detection of neutralizing antibody titers to HPV types 4, 38, 75, 76, and 96 in guinea pig sera that were previously negative in the standard L1-PBNA (Fig. 7b).

### PfTrx-L2c12merOVX313 and PfTrx-L2c14merOVX313 induce the production of neutralizing antibodies affording in vivo protection in a mouse challenge model

**Fig. 8:**
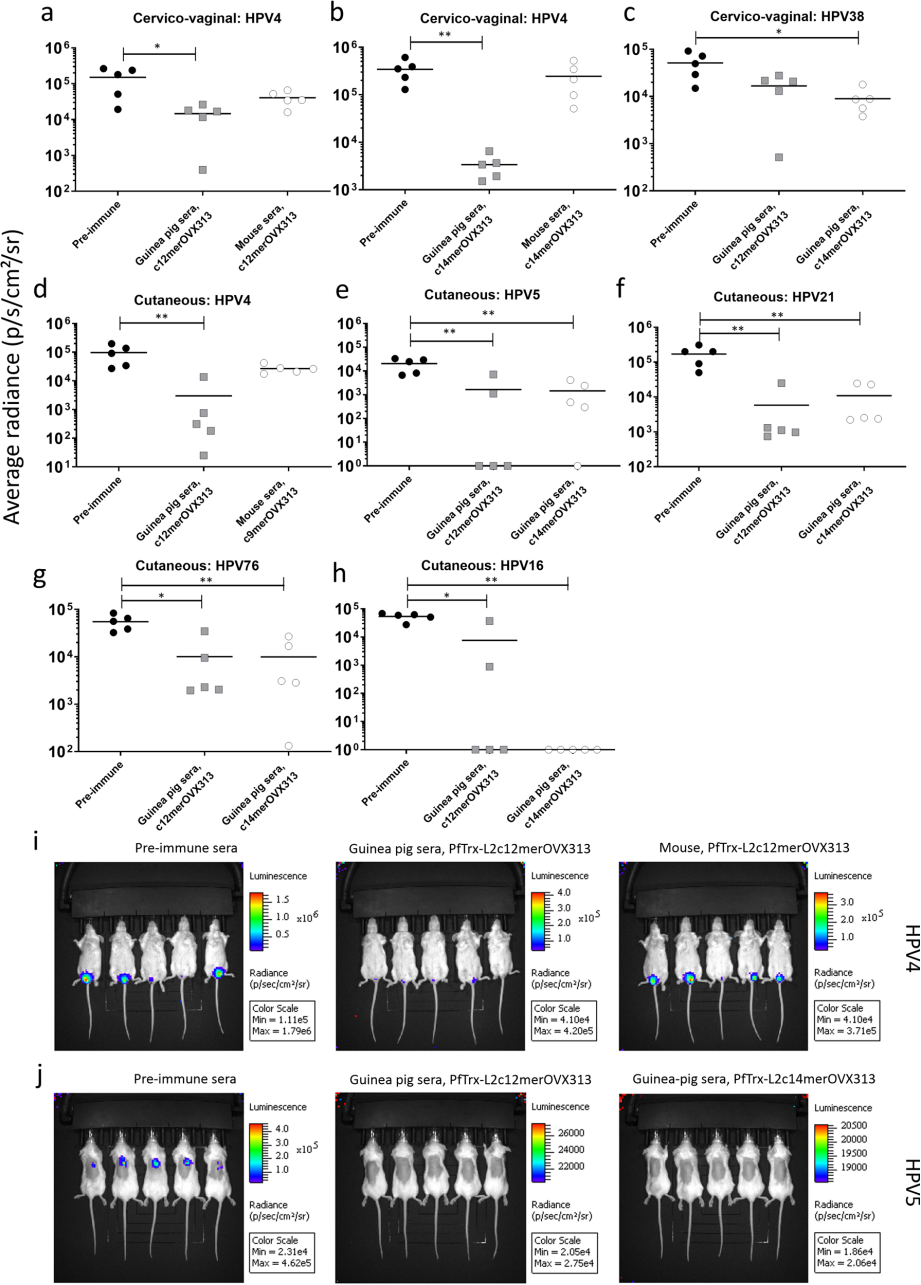
Sera raised against the leading candidate vaccines passively protect naïve mice against HPV PSV challenge. Pools of sera derived from either 10 mice, or 2 guinea pigs immunized with PfTrx-L2c9merOVX313 (20 µg), PfTrx-L2c12merOVX313 (30 µg) or PfTrx-L2c14merOVX313 (30 µg) were passively transferred to naive mice. Protection against cervico-vaginal (**a**–**c**) or cutaneous challenge (**d**–**h**) with the indicated cutaneous HPV PSV types was compared to passive immunization with sera from non-immunized mice (*pre-immune*). The *y*-axis represents the average radiance measured as photons per second per square centimeter per steradian. Note that different scales were employed in order to take into account the different in vivo transduction efficiencies of individual HPV PSV types. A *P*-value of ≤0.05 was considered significant. *P < 0.05; **P < 0.01; ***P < 0.001, as determined by the nonparametric Mann–Whitney test. Representative images of the passive transfer of immune sera followed by cervico-vaginal (**i**) or cutaneous challenge (**j**) with the indicated cutaneous HPV PSV types. Colors (scale shown on the right) represent the intensity of luciferase expression upon transduction and challenge with the indicated PSVs as average radiance (photons per second per square centimeter per steradian).

Next, we used two different mouse challenge models to assess vaccine-induced protection via passive transfer of immune sera, focusing on three subsets of cHPV types: (i) HPV types 4, 38, and 76 against which, rather weak antibody titers were previously detected with the L1-PBNA; (ii) HPV types 5 and 21 against which, relatively strong antibody titers were found; (iii) HPV16, representing high-risk mucosal types to which antibody titers were not characterized. In this experimental set-up, passively transferred sera of guinea pigs immunized with the PfTrx-L2c12merOVX313 antigen proved capable of significantly reducing the PSV transduction signal derived from the cervico-vaginal challenge with HPV4 (Fig. 8a, i) and cutaneous challenge with HPV4, HPV5, HPV21 and HPV76 (Fig. 8d–g, j and Supplementary Fig. 2), in comparison to the pre-immune sera. A reduced mean average radiance was also observed upon cervico-vaginal challenge with HPV38, but this was not statistically significant (mean average radiance of 50,000 p/s/cm²/sr for the pre-immune sera, compared to 16,000 p/s/cm²/sr for the sera from PfTrx-L2c12merOVX313-immunized mice, *p*-value = 0.055 as determined by the nonparametric Mann–Whitney test; see Fig. 8c and Supplementary Fig. 3). By comparison, passive immunization with sera from guinea pigs immunized with PfTrx-L2c14merOVX313 afforded significantly reduced transduction upon cervico-vaginal challenge with HPV4 and HPV38 (Fig. 8b, c, respectively, and Supplementary Fig. 3), and upon cutaneous challenge with HPV5, HPV21, and HPV76 (Fig. 8e–g, and Supplementary Fig. 2), in comparison to the pre-immune sera.

Under similar experimental conditions, mouse sera raised against *Pf*Trx-L2c12merOVX313 afforded a lower mean average radiance in animals challenged intra-vaginally with HPV4 (mean average radiance of 150,000 p/s/cm²/sr for the pre-immune sera, compared to 40,000 p/s/cm²/sr for pooled sera from mice immunized with *Pf*Trx-L2c12merOVX313, *p*-value = 0.222 as determined by the nonparametric Mann-Whitney test) (Fig. 8a, i), although not at statistically significant levels, whereas sera from mice immunized with PfTrx-L2c14merOVX313 yielded a similar mean average radiance to that of the pre-immune sera (mean average radiance of 245,000 p/s/cm²/sr for the pooled immune-sera, compared to 342,000 p/s/cm²/sr for the pre-immune sera, *p*-value = 0.309 as determined by the nonparametric Mann–Whitney test; Fig. 8b and Supplementary Fig. 3). Interestingly, mouse sera raised against PfTrx-L2c9merOVX313, which were all found to be non-neutralizing to HPV4 by the standard L1-PBNA (Fig. 5), reduced the PSV transduction signal upon cutaneous challenge with the same cHPV type (mean average radiance of 26,000 p/s/cm²/sr for the pooled immune-sera, compared to 97,000 p/s/cm²/sr for the pre-immune sera, *p*-value = 0.05 as determined by the nonparametric Mann–Whitney test) (Fig. 8d and Supplementary Fig. 2). Collectively, these findings confirm the in vivo immunogenicity of our leading candidate vaccines against multiple cutaneous HPV types, previously predicted by in vitro neutralization assays.

### PfTrx-L2c12merOVX313 as the overall most promising cHPV vaccine candidate

Multiple factors, including ease of production and scalability, influence vaccine developability and potential for translation to a clinical setting. The immunogenicity of PfTrx-L2c12merOVX313 and PfTrx-L2c14merOVX313 antigens were found to be overall quite comparable. In guinea pigs, the latter antigen-induced neutralizing antibodies against 18, rather than 16 cHPV types, but the resulting sera failed to provide protection against cutaneous challenge with HPV4 and a similarly inferior protection compared to PfTrx-L2c12merOVX313 was observed with mice immune-sera. However, an important favorable feature of the 12mer antigen was its higher production yield and ease of purification. In fact, PfTrx-L2c12merOVX313 not only was expressed at higher levels compared to 14mer antigen, but it was also more amenable to a GMP-compatible, heat-treatment and cation-exchange chromatography-based purification, with a final yield of homogenously purified protein (>9 mg/l of bacterial culture) almost 10-fold higher than that achieved with PfTrx-L2c14merOVX313 (Supplementary Fig. 4).

**Fig. 9:**
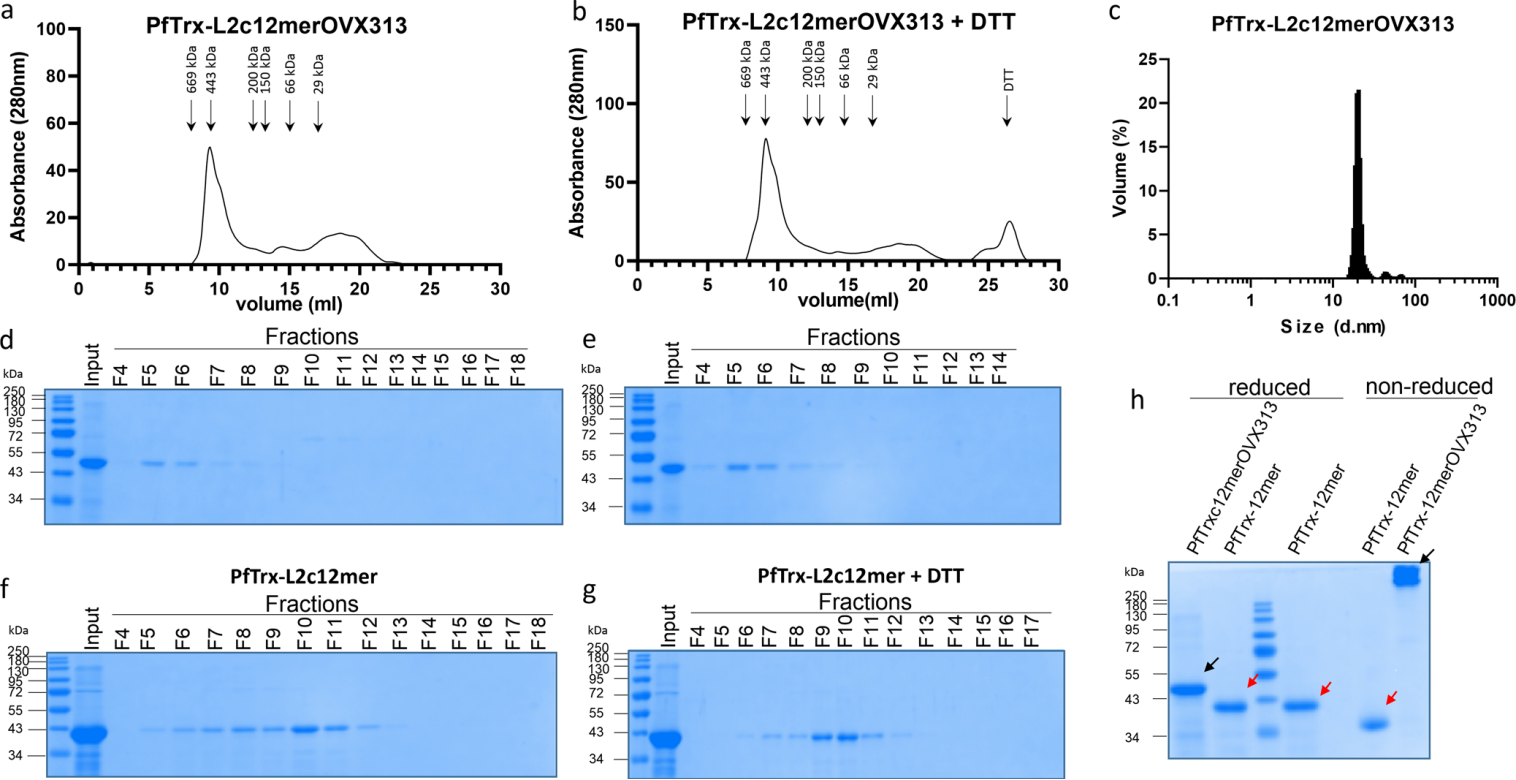
OVX313 leads to a stable oligomeric form of the PfTrx-L2c12mer antigen. **a**, **b** Size-exclusion chromatography (SEC) profiles of purified PfTrx-L2c12merOVX313. **c** Dynamic light scattering analysis of the PfTrx-L2c12merOVX313 antigen indicates the presence of monodisperse nanoparticles with a diameter of 20 nm. **d**, **e** SEC fractions (**a**, **b**) analyzed by SDS-PAGE. **f**, **g** OVX313-lacking PfTrx-L2c12mer antigen purified by SEC and analyzed by SDS-PAGE. **h** The purified PfTrx-L2c12merOVX313 protein (black arrow) and its monomeric, OVX313-lacking PfTrx-L2c12mer form (red arrow) analyzed by SDS-PAGE under reducing and non-reducing conditions.

Furthermore, the purified PfTrx-L2c12merOVX313 antigen behaved as a single, soluble and monodispersed protein species when analysed by size exclusion chromatography (Fig. 9a, b) and dynamic light scattering under native conditions (Fig. 9c), with an apparent molecular mass of 450 kDa and an estimated molecular diameter of 20 nm. These features were not observed for an OVX313-lacking PfTrx-L2c12mer antigen, which assembled into a less uniform, aggregated state not as resistant to DTT treatment as its PfTrx-L2c12merOVX313 counterpart (Fig. 9d–g). Supporting these findings, we found that the OVX313 domain leads the PfTrx-L2c12merOVX313 antigen to assemble into higher molecular weight structures when proteins are analysed in SDS-PAGE gel under non-reducing conditions, which is otherwise not observed in its OVX313-lacking PfTrx-L2c12mer form (Fig. 9h).

Although the OVX313 domain, with its cluster of five sequential C-terminal arginine residues, clearly contributes to a superior production/purification performance, other factors, likely related to the larger size of the incorporated polytope, are probably responsible for the suboptimal production yield and less straightforward purification of the PfTrx-L2c14merOVX313 antigen. Altogether, our findings clearly point to the feasibility of a scalable production of the PfTrx-L2c12merOVX313 vaccine candidate under GMP-compliant conditions.

## Discussion

The use of L1 VLP-based immunogens to target diverse cutaneous HPV types is hindered by the type-specific antibody response induced by the L1 conformational epitopes responsible for virus neutralization. Development of a multivalent VLP-based vaccine against cutaneous HPV types of clinical relevance for immunosuppressed individuals is ultimately a technical hurdle given the immense variety of genotypes to be covered. Conversely, the papillomavirus minor capsid protein L2 harbors highly conserved regions that can induce neutralizing responses against homologous and heterologous viral types^41^. In particular, the aa 20–38 region we employed as immune-epitope in our vaccine design^37^, contains two cysteine residues (22 and 28) that are conserved within the L2 proteins of all known papillomaviruses. The display of L2 aa 17–36 region (‘RG1’ epitope) into chimeric HPV VLPs was shown to induce cross-neutralizing responses in rabbit^42^. Nevertheless, an L2 epitope from a single HPV type does not provide a sufficiently comprehensive coverage^43,44^. For this reason, we employed a polytope design for comprehensive coverage of a large panel of different HPV types.

Here, we demonstrated that our leading vaccine candidate, PfTrx-L2c12merOVX313, consistently induces neutralizing antibody responses against eight out of eleven tested cutaneous HPV types in mice. An even more favorable picture emerged from immunization studies in guinea pigs, with the induction of neutralizing antibodies against 19 (out of 21) tested cutaneous HPV types. It is also important to note that neutralizing antibody titers measured with the L1-PBNA likely underestimate the actual humoral responses induced by the cHPV vaccine prototypes. This view is supported by the results we obtained with the more sensitive FC-PBNA^45^, which revealed significantly increased neutralizing antibody titers against all the five tested HPV types. Although generally considered as the gold standard for measuring functional antibody responses to papillomaviruses, the L1-PBNA does not closely mimic the early stages of infection that occur in vivo. These early infection stages are critical for the proteolytic processing of the viral capsid, which leads to the exposure of the cross-neutralization epitopes comprised within the N-terminal region of L2. Consequently, the L1-PBNA has a suboptimal sensitivity for the detection of L2-targeting neutralizing antibodies. Furthermore, the in vivo functionality of the neutralizing antibodies elicited by the cHPV vaccine leads was confirmed by their ability to afford partial or complete reduction of HPV-derived radiance signal via cervico-vaginal and cutaneous challenge in naïve mice, even for cHPV types whose neutralizing antibody titers measured with the L1-PBNA were rather low or barely detectable. Importantly, none of these in vitro neutralization assays capture protection afforded by antibody-dependent phagocytosis, a mechanism recently identified for L2 antibody-mediated responses^46^.

Comprehensively strong immune responses against many cutaneous and even mucosal HPV types were induced by the monomeric PfTrx-L2c6mer and PfTrx-L2c9mer antigens in guinea pigs, but the same antigens proved to be surprisingly weak in mice. This yet unexplained immunogenicity gap was partially compensated by antigen reformulation through genetic fusion to the OVX313 oligomerization domain, which has been previously applied successfully to the PANHPVAX vaccine^38^. Oligomeric reformulation yielded monodipersed heptameric nanoparticles, which could be purified to high homogeneity by a thermal treatment (80 °C) followed by a single chromatographic step. We believe that oligomeric epitope display, plus the presence of additional T-helper epitopes associated to the OVX313 heptamerization domain, are responsible for the enhanced immunogenicity of the OVX313-conjugated antigens^38,39^. The modular nature of our approach was thus instrumental to the identification of what we believe is the best compromise between antigen complexity and multi-immunogenicity. For example, despite the inclusion of the OVX313 domain, the simpler PfTrx-L2-c6mer antigen elicited a fairly weak anti-HPV41 response—much lower than that afforded by the more complex PfTrx-L2c9merOVX313 antigen. In this regard, the HPV41 L2 epitope seemed essential for inducing type-specific neutralization in the context of our vaccine platform. Similar results were obtained in the case of HPV63, with an additional interesting aspect: cross-neutralizing antibody titers to HPV63 were likely induced by the HPV49 L2 epitope, only present in the PfTrx-L2c14merOVX313 antigen. Supporting our assumption is the fact that both L2 epitopes display two amino acids residues—Ala and Asn—at the corresponding position, in a very unique manner among all other epitopes explored. This highlights how a single epitope can critically contribute to the immune-performance of our modular vaccine design. However, the increased broadness of protection afforded by high-complexity antigens such as PfTrx-L2c14merOVX313 may come at the expense of lower recombinant protein expression levels and a less straightforward purification process. This kind of balance between immunogenicity and ease of production makes us envisage PfTrx-L2c12merOVX313, which can be obtained with a relatively high yield under GMP-compatible production conditions, as the leading candidate for a broad-coverage vaccine against cutaneous HPVs.

Seminal research on papillomavirus has demonstrated that relapses from warts causally associated to HPV1 are linked to decreased humoral immune responses^47^, suggesting a protective role of cHPV-specific antibodies against cutaneous lesions. Vaccine-mediated prevention against experimentally induced skin infection caused by papillomavirus has been demonstrated in rabbits^48^, cattle^49^, and horses^50^. A substantial body of epidemiological data has linked cutaneous HPV infection to squamous cell carcinoma^6,25,26,51–54^, a neoplastic pathology that is particularly aggressive in transplanted patients^55,56^. The most promising evidence supporting a protective role of vaccination against papillomavirus-induced skin cancer was obtained by Vinzón et al.^57^. In this work, the authors demonstrated that *Mastomys coucha* mice—a rodent species that is naturally susceptible to the development of skin tumors caused by *Mastomys natalensis* papillomavirus (MnPV) infection—were protected against benign and malignant skin tumors by anti-MnPV L1 VLP vaccination, both under normal as well as immunosuppressive conditions. In line with these findings, and in keeping with the predicted broadness of protection afforded by our multiepitope cHPV vaccine design, we have recently found that immunization of virus-free *M. coucha* with PfTrx-L2c12merOVX313 elicits the production of neutralizing antibodies leading to protection against MnPV-induced cutaneous tumors (Ahmels et al., submitted). Despite this fairly strong circumstantial evidence, final proof that antibodies are preventive against HPV-induced skin cancer in healthy and immunocompromised individuals will only be obtained from clinical trials in humans.

It has been proposed that the carcinogenic role of cutaneous HPVs in skin cancer relies on the virus-promoted accumulation of ultra-violet-induced mutations in essential tumor suppressor genes (e.g. p53^58^) within cells located in sun-exposed body sites. Cells thus transformed would no longer depend on cutaneous HPV infection for progression toward malignant lesions, a carcinogenesis model named ‘hit-and-run’^15^. This model has recently been challenged by Strickley et al.^59^, who proposed that the loss of T-cell immunity against commensal papillomaviruses, rather than a virus-induced pro-oncogenic effect, is the cause of increased risk to develop skin cancer. In our opinion, conclusive evidence for a causative involvement of cutaneous HPVs in skin cancer could only emerge from vaccination studies, assuming a preventive effect by the vaccines against tumor formation.

The major limitations of our study are related to (i) the restricted number of cutaneous HPV types evaluated by the in vivo murine challenge model; (ii) the low production yields of the PfTrx-L2c14merOVX313 antigen; and (iii) the lack of a demonstrated vaccine-induced immune-reactivity against some cutaneous HPV types targeted by the antigens (e.g. HPV15, HPV49, and HPV88). The first limitation is mainly due to the technical difficulties in establishing consistently measurable PSV infections at cutaneous sites. However, the comprehensive number of cutaneous and mucosal HPV types tested with our PBNA panel partially compensates for this limitation. Regarding the second point, and in view of our goal to design a low-cost, broadly protective vaccine, we reasoned that despite its slightly lower performance compared to PfTrx-L2c14merOVX313, the immunogenicity profile displayed by the PfTrx-L2c12merOVX313 antigen meets the requirements of a broadly protective vaccine. The third limitation, namely the production of sufficiently active cutaneous HPV pseudovirions, has to do with a still unsolved technical challenge experienced by different laboratories^42^. For HPV1, HPV2 and HPV41, we circumvented this problem through the production of genetically engineered, infective ‘hybrid’ PSVs displaying the corresponding heterologous L2 (aa 20–38) epitopes on the capsid surface of HPV3, but this approach was not successful with other HPV types. Although artificial, the use of ‘hybrid’ PSVs produced results well correlated with the expected immunogenicity of our vaccine candidates. Nevertheless, caution is needed in interpreting immunogenicity data related to these particular HPV types. A minor limitation refers to the lack of data on the durability of neutralizing antibody responses induced by our lead candidate. Nevertheless, we have demonstrated that neutralizing antibody levels induced by the PANHPVAX are measurable for up to 1-year after immunization^60^, and we suspect this may also be the case for our cutaneous candidate vaccines.

In conclusion, we document the development of a broadly immunogenic prototype vaccine against cutaneous HPV types associated to post-transplantation skin lesions in organ-transplanted patients. Our leading vaccine candidate, PfTrx-L2c12merOVX313, also appears to be well suited to a GMP-compatible production process—an additional important prerequisite for entering a clinical study. It should also be noted, in this regard, that PANHPVAX, the L2 polytope-based mucosal HPV vaccine that shares the same scaffold components (PfTrx and OVX313) as our lead cHPV vaccine candidate, will enter into a phase I clinical evaluation mid of 2022. Because of such compositional similarity, this phase I study will thus also provide indirect but important information on the safety, and prospective in-humans efficacy, of PfTrx-L2c12merOVX313. In this respect, PANHPVAX has been successfully manufactured under GMP and GMP protocols could be readily adapted to PfTrx-L2c12merOVX313. A prophylactic vaccine capable of protecting organ transplant recipients against HPV-related skin lesions should induce a broad-spectrum immune response. We propose a strategy of immunization prior to iatrogenic immunosuppression based on the observations that OTRs are at greater susceptibility risks to acquire skin lesions presenting multiple HPV types following transplantation^10,24^. The prevalence of skin warts also increases with time since transplantation^61^. Whether this is due to recrudescence or new HPV infections remains to be demonstrated. It should be noted, likewise, that the skin colonization by HPV is a very dynamic process, which shows distinct age- and sex-dependent seroprevalence patterns for phylogenetically distinct genera. Antibodies to cutaneous mu- and nu HPV types appear early in life whereas those to beta- and gamma cutaneous types are rare in children and accumulate later in life (>40 years in women, >50 years in man)^62^. Therefore, children, adolescents, young-, middle-aged and older adults electable for organ transplantation are all at higher risks of developing skin lesions, and may have different windows of opportunity to benefit from a vaccine targeting cutaneous types. What is of utmost importance, in our opinion, is the understanding that the value of a cutaneous HPV vaccination for preventing post-transplantation skin lesions can only be evaluated in clinical studies with OTRs of different ages, before the onset of immunosuppression, mainly due to the knowledge gaps and shortcomings on the role of cutaneous HPV in skin lesions. A similar vaccination protocol is already in place for other vaccines administered to OTRs such as measles, mumps, rubella, varicella, pneumococcus, influenza, hepatitis B, meningococcal and other vaccines^63^. Assuming a protective effect against skin lesions, vaccination protocols can be further adapted (i.e. as a routine in the childhood) according to the follow-up studies, as still happens for the currently available HPV vaccines targeting anogenital cancers (previously administered to 16–17 years girls upon a 3-dose regimen, currently recommended for 9–11 years boys and girls upon a 2-dose regimen). A vaccine specifically targeting cutaneous HPVs would thus represent an important prophylactic addition, but it could also shed light on the causative role of cHPVs in skin carcinogenesis.

## Methods

### Antigen design and purification

Putative cross-neutralizing epitopes comprising 19 residues found in the N-terminus of the L2 protein of several cutaneous HPV types were predicted by sequence homology to the HPV16 L2 amino acid (aa) 20–38 epitope previously characterized by us^37^ (for illustration, see Supplementary Fig. 1A). According to clinical relevance and amino acid sequence homology, six to fourteen of these cutaneous HPV-derived L2 aa20–38 epitope homologs were grouped in silico in multimeric polytopes and inserted into the N-terminus of the *P. furiosus* Trx coding sequence. The resulting PfTrx-L2 polytopes (c6mer, c7mer, c9mer, c10mer, c12mer, and c14mer) were further C-terminally fused to the OVX313 oligomerization domain, a derivative of the complement inhibitor C4-binding protein^64,65^. Codon-optimized genes coding for eight antigens were chemically synthetized (GenScript) and genetically inserted into the NdeI-BamHI site of pET24b expression plasmid. The resulting constructs were verified by sequencing and transformed into electro-competent *Escherichia coli* BL21 codon plus (DE3) cells.

Recombinant protein expression of the different antigens were typically assessed in small-scale (4 ml) overnight cultures (LB medium at 30 °C) in the presence of 2 mM IPTG. Upon confirmation of expression via electrophoresis in Coomassie-blue stained SDS-PAGE gels, protein production was scaled-up to 400 ml bacterial cultures, followed by cell harvesting and washing, and lysis via high pressure (French press) in the presence of 25 mM Tris, 300 mM NaCl, 0.1 mg/ml lysozyme, 1 mM PMSF, 0.16% Tween 20 at pH 8. Following centrifugation of bacterial total lysate, the supernatant (cleared lysate) generally underwent a heat treatment (incubation at 65–85 °C for 30 min) that yielded considerably pure (>80%) protein preparations, given the extreme thermo-stability of the PfTrx scaffold protein. For the OVX313-fused versions of the antigens, we developed an additional cation-exchange chromatography step through which the protein samples were loaded onto a HiTrap sepharose column (SP fast-flow, GE) equilibrated in 0.2 M NaCl-containing PBS, pH 8. The target antigens were eluted via a saline gradient (0.2–1 M NaCl-contaning PBS buffer). Individual peak fractions monitored by SDS-PAGE were pooled, exchanged into PBS pH 7 and quantified via SDS-PAGE against a curve of standard BSA. The spontaneous oligomerization of OVX-fused antigens was evaluated by SDS-PAGE under non-reducing conditions, size-exclusion chromatography and dynamic light scattering as described below. The final protein samples were detoxified as follows: 1% (v/v) Triton X-114 was added to each protein prep and the mixture was incubated on ice for 5 min, and then for additional 5 min at 37 °C. Next, the samples were centrifuged at 10,600 rcf for 1 min at 37 °C^66^. The resulting translucent supernatant was carefully collected in order to avoid contamination by the opaque pellet formed by micelles pelleted down in the tube. The procedure was repeated once, and the final supernatant aliquots were then stored at −20 °C.

Production and purification of leading candidates was also assessed in proprietary minimal BMM (https://www.dsmz.de/microorganisms/medium/pdf/DSMZ_Medium242.pdf) or in soya-supplemented BMM (SBMM).

### Animal immunization

Six- to eight-weeks-old female BALB/c mice were purchased from Charles River Laboratories (Sulzfeld, Germany) and kept under specific-pathogen-free conditions (animal permit G248/16, Regierungspräsidium Karlsruhe, Germany). We have previously demonstrated that the adjuvantation of our candidate vaccines is necessary to induce strong L2 neutralizing antibody responses against mucosal HPV types^38^. Moreover, the use of AddaVax (InvivoGen), a MF59-like a squalene-based, oil-in-water nanoparticle emulsion afforded robust L2 neutralizing antibody responses when formulated with our PfTrx-L2-OVX313 prototype antigen targeting HPV16^40^. Therefore, we employed AddaVax as adjuvant for the cutaneous HPV candidate vaccines explored in this current pre-clinical study. All mice were immunized four times at biweekly intervals with intramuscular injections of 50 µl doses containing 20 µg of the antigens adjuvanted with 50% (v/v) AddaVax (InvivoGen). Four weeks after the last dose, the mice were sacrificed and blood samples obtained via heart puncture. 150–200 g outbread Hartley (Crl:HA) female guinea pigs (Charles River; Sulzfeld, Germany) (animal permit A2/17, Regierungspräsidium Karlsruhe, Germany) were immunized subcutaneously four times at triweekly intervals with 100 µl doses containing 30 µg of the antigens similarly adjuvanted with AddaVax. Collection of blood samples proceeded as described for mice.

### HPV pseudovirion preparation

Cutaneous HPV pseudovirions (PSV) were used as virus surrogate during our in vitro neutralization assays and murine challenge models (Supplementary Table 7). Human codon usage-optimized bicistronic DNA constructs containing a Kozak sequence and coding for the L1 and L2 capsid genes (based on accession numbers retrieved from the Papillomavirus Episteme, PaVE - https://pave.niaid.nih.gov/) of 18 cutaneous HPV types were synthetized (GenScript) and cloned downstream of a CMV promoter (p16shell plasmid^67^, ClaI-HindIII sites for L1, NotI-XbaI sites for L2). The L1-L2 bicistronic region contains an IRES sequence between the capsid genes, and a bovine growth hormone poly(A) tail. PSV constructs for HPV1, HPV2, and HPV41 were designed differently: the nucleotide sequence encoding the HPV3 L2 aa 1–46 was replaced by the corresponding HPV1, HPV2, and HPV41 L2 regions, giving rise to ‘hybrid’ HPV3 PSV displaying those grafted L2 aa20–38 epitopes. All PSV constructs designed for this work were confirmed by sequencing. The following PSV constructs were kindly provided by colleagues: p5shell from Dr. John Schiller (National Cancer Institute, Bethesda, USA); p38shell and p76shell from Dr. Joakim Dillner (Karolinska Institutet, Sweden); p92shell and p96shell from Dr. Reinhard Kirnbauer (Medical University Vienna, Austria). Nucleotide sequences of those constructs are available at https://ccrod.cancer.gov/confluence/display/LCOTF/Home.

Human embryonic kidney cells (HEK293TT; 3 × 10^6^ cells) were seeded in 10 ml of DMEM supplemented medium per 10 cm tissue culture dish (10 dishes were used per PSV prep) and co-transfected 16 h later with 15 µg of a HPV-related construct plus a reporter plasmid coding for a Gaussia-luciferase protein (GL), using polyethylenimine (PEI, Sigma-Aldrich). Cells were incubated for 3 days in a humidified incubator at 37 °C in the presence of PEI/DNA complexes. Transfection efficiency was monitored by fluorescence microscopy, as the GL-reporter plasmid also encodes a green-fluorescent protein (GFP). At the third day of transfection, cells were harvested from the culture dishes, transferred to 50 ml conical tubes and centrifuged for medium removal. After washing the cells in PBS and transfer to 2 ml low-binding tubes (Sigma-Aldrich), a new centrifugation took place in order to pellet the cells. For each milligram of cell pellet harvested, 1 µl of DPBS supplemented with 10% Brij58 (v/v) (Sigma-Aldrich) and 0.1% RNAse cocktail (v/v) (Thermo Fischer) was used to re-suspend the cells by inversion. Incubation of cell lysate proceeded for 2 days at 37 °C in an end-over-end rotator for maturation of PSV. Then, the lysate was chilled on ice for 5 min and salt concentration of cell lysate was adjusted to 850 mM by adding 0.17 volumes of 5 M NaCl. Following an additional incubation on ice for 10 minutes, the lysate was cleared by centrifugation (10.600 rcf, 10 min at 4 °C) and transferred to a new low-binding tube placed on ice. DPBS/0.8 M NaCl was used to wash and extract PSV particles loosely bound to the pellet obtained after centrifugation of the lysate (1 µl of buffer per milligram of pellet). After centrifugation, both cleared lysates were combined and incubated with 2.5 units/ml turbo nuclease (Jena Bioscience) for 1 h at 37 °C.

Purification of PSV particles from the cleared lysate was carried out by ultracentrifugation through a 27, 33, and 39% Optiprep step gradient for 5 h at 16 °C at 234,000 rcf at acceleration 9 and deceleration 1. After ultracentrifugation, 0.5 ml gradient fractions were collected in 1.5 ml low-binding tubes and screened for transduction activity in cell culture. For this, aliquots of each gradient fraction were diluted 1:1000 with DMEM supplemented medium and added to 12.5 × 10³ HeLa TK4 cells^68^. This mixture was prepared in duplicates using 96-well plates further incubated at 37 °C for 2 days in a humidified incubator. Following incubation, PSV-transduced GL is secreted into the medium and its concentration was determined in 10 µl of cell culture medium using the coelenterazine substrate and Gaussia glow juice (PJK, Germany), according to the instructions of the manufacturer. A microplate luminometer (Envision 2101 multi-label reader, PerkinElmer) was used to determine the culture medium associated luminescence after 15 min of substrate addition. Confirmation of PSV-derived transduction was achieved by a combination of polyclonal and monoclonal neutralizing antibodies. PSV infectivity was determined based on the fraction dilution yielding 100-fold higher relative luminescent units (RLU) as compared to its control in the presence of a neutralizing antibody.

The production of furin-cleaved-(fc-) PSV was carried out in a furin-overexpressing 293TTF producer cells^45^. In brief, PSVs are subjected to maturation in the presence of furin, which proteolytic activity has been demonstrated to be a critical step for HPV infectivity. Furin cleaves L2 at its N-terminus allowing exposure of the targeted cross-neutralization epitopes and further binding to secondary cell receptors before infection. Following purification as described above, the transducibility of fc-PSV particles is then measured in furin-deficient LoVoT reporter cell line, which is permissive to fc-PSV transduction while insensitive to non-cleaved PSV ones.

For the in vivo HPV challenge models, PSV particles carrying a firefly-luciferase (FFL)-encoding plasmid were produced and purified as described above. The intracellular FFL transduction was measured with Beetle-Juice luciferase assay (PJK, Germany), according to the manufacturer’s recommendations.

### HPV pseudovirion-based neutralization assay (PBNA)

Neutralizing antibody titers were determined by the standard PBNA (so-called L1-PBNA)^68^. Briefly, serum samples were serially diluted six times in a 96-well plate using a threefold increment to achieve a final serum dilution in the neutralization assay of 1:50 to 1:12,000. Next, the PSV previously diluted in DMEM were added to the diluted sera and the mix incubated for 20 min at room temperature. Finally, HeLa TK4 (10^6^ cells) were added to the PSV-serum mixture and the assay plate incubated at 37 °C for 2 days in a humidified incubator. PSV-transduced GL activity was determined as described above. All sera were tested in duplicates. The serum neutralization activity (% inhibition) was calculated via the GraphPad software program by normalizing the luciferase signal measured for ‘PSV-serum’ against the corresponding signal for ‘PSV-alone’. Neutralizing antibody titers (EC50) represent the serum dilution in which a 50% inhibition of PSV-induced luciferase signal is seen. EC50 values <50 were considered non-neutralizing and therefore set to 0.1.

An alternative assay named FC-PBNA^45^ and employing fc-PSV particles was also deployed to complement our analysis. This assay is better suited to the detection of L2-raised neutralizing antibodies as a result of the improved display of targeted aa 20–38 cross-neutralization epitopes following furin cleavage of the HPV capsid. Neutralization of fc-PSV particles is assessed with the use of the furin-deficient LoVoT reporter cell line. The assay layout is similar to the L1-PBNA.

### In vivo HPV challenge

Six to eight-week-old female BALB/c mice were obtained from Charles River Laboratory (animal permit G93/19, Regierungspräsidium Karlsruhe, Germany) and subjected to a cervico-vaginal PSV challenge^38^. For this, male mouse cage bedding was transferred to groups of female mice for inducing hormonal synchronization (Whitten effect, day 1). Two days later, 3 mg of medroxyprogesterone acetate (Depo-Provera, Pharmacia) were administered subcutaneously. On the following day, each mouse was injected intraperitonealy with 100 µl of a pool of immune sera (obtained from immunized animals and diluted 1:2 in 1× PBS). One day after passive transfer of sera (day 5), the mice were treated intravaginally with 50 µl of 4% nonoxynol-9 (N9, Spectrum) in 4% carboxymethyl cellulose (CMC, Sigma). Four hours later on the same day, HPV PSV in 4% CMC was instilled intravaginally. The in vivo PSV-derived transduction was measured two days later by instilling 20 µl luciferin substrate (15 mg/ml, Promega) intravaginally and using an in vivo imaging system (IVIS) imager (Xenogen Corporation, PerkinElmer). A region-of-interest (ROI) around the luminescence emitting area of each mouse was obtained using the Living Image 2.50.1 software (Xenogen, PerkinElmer) to determine the average radiance (photons per second per square centimeter per steradian, p/s/cm²/sr). The background signal was obtained by imaging each group of mice prior to instillation of luciferin, and subtracted from the average radiance obtained post-instillation.

For the cutaneous PSV challenge, the animals had its dorsal hair removed with an electric clipper followed by application of a depilatory cream for 20 s. Under anaesthetic, an area of 2 × 1.5 cm of the shaved region was carefully scraped using a scalpel to remove the uppermost layers of the epidermis without causing cuts or bleeding. Two days later, 20 µl of cutaneous PSV in 0.6% CMC (1:2) was added to the scrapped region. The luminescence read-out was assessed 2 days post-infection by dropping 30 µl of luciferin substrate onto the scarified region. Determination of average radiance was carried out as described for the cervico-vaginal challenge model.

### Size-exclusion chromatography and dynamic light scattering

A Superdex 200 Increase 10/300 GL column (GE Healthcare) equilibrated with PBS at a flow-rate of 0.5 ml/min was employed for size-exclusion chromatography (SEC). The column was applied in an Äkta fast protein liquid chromatography (FPLC) system, using UV 280 nm wave-length and 1.5 MPa as the maximum pressure, carried out with a Unicorn 5.0 software (Amersham, GE Healthcare). Protein elutions were obtained with 2 column volumes of running buffer. Fractions of 1 ml were collected and stored at 4 °C until further analysis by electrophoresis in SDS-PAGE gel and coomassie dye-binding assay. A Zetasizer Nano ZM device (Malvern, United Kingdom) was employed for analysis of the hydrodynamic sizes of nanoparticle antigens by dynamic light scattering (DLS).

### Statistics

HPV type-specific seroprevalence (proportion of neutralizing antibody-neutralizing samples of all serum samples) with 95% confidence intervals (using the method of Clopper-Pearson), and mean neutralizing antibody titers with standard error of mean were calculated using GraphPad 8.3.1. Statistical significance was determined with the nonparametric Mann-Whitney test, also via GraphPad.

### Reporting summary

Further information on research design is available in the Nature Research Reporting Summary linked to this article.

## Supplementary information

Supplementary Material

REPORTING SUMMARY

## Data Availability

The authors confirm that the raw data of this study were generated at the DKFZ. In addition to the Supplementary Tables and Supplementary Figures, further data supporting the findings of this study are available upon reasonable request to the corresponding authors according to DKFZ data safety protection regulations.

## References

[1] Human papillomaviruses. *IARC Monographs on the Evaluation of Carcinogenic Risks to Humans* **90**, 1–636 (2007). PMC4781057 18354839

[2] de Martel C, Plummer M, Vignat J, Franceschi S (2017). Worldwide burden of cancer attributable to HPV by site, country and HPV type. Int. J. Cancer.

[3] Bruni L, A. G. et al. *Human Papillomavirus and Related Diseases in the World*. (ICO/IARC Information Centre on HPV and Cancer (HPV Information Centre), 2022).

[4] Van Doorslaer K (2011). Papillomaviruses: evolution, Linnaean taxonomy and current nomenclature. Trends Microbiol..

[5] de Koning MNC (2009). Prevalence and associated factors of betapapillomavirus infections in individuals without cutaneous squamous cell carcinoma. J. Gen. Virol..

[6] Iannacone MR (2014). Case-control study of genus-beta human papillomaviruses in plucked eyebrow hairs and cutaneous squamous cell carcinoma. Int. J. Cancer.

[7] Ma Y (2014). Human papillomavirus community in healthy persons, defined by metagenomics analysis of human microbiome project shotgun sequencing data sets. J. Virol..

[8] Pfister, H. Chapter 8: Human papillomavirus and skin cancer. *J. Natl Cancer Inst. Monogr.* 52–56 (2003). 10.1093/oxfordjournals.jncimonographs.a003483 12807946

[9] Bzhalava D (2014). Deep sequencing extends the diversity of human papillomaviruses in human skin. Sci. Rep..

[10] Euvrard S, Kanitakis J, Claudy A (2003). Skin cancers after organ transplantation. N. Engl. J. Med..

[11] Henley JK, Hossler EW (2017). Acquired epidermodysplasia verruciformis occurring in a renal transplant recipient. Cutis.

[12] Karia PS, Han J, Schmults CD (2013). Cutaneous squamous cell carcinoma: estimated incidence of disease, nodal metastasis, and deaths from disease in the United States, 2012. J. Am. Acad. Dermatol..

[13] de Jong E, Lammerts M, Genders RE, Bouwes Bavinck JN (2022). Update of advanced cutaneous squamous cell carcinoma. J. Eur. Acad. Dermatol. Venereology.

[14] Bouwes Bavinck JN (2007). Keratotic skin lesions and other risk factors are associated with skin cancer in organ-transplant recipients: a case-control study in The Netherlands, United Kingdom, Germany, France, and Italy. J. Investigative Dermatol..

[15] Hasche D, Vinzon SE, Rosl F (2018). Cutaneous papillomaviruses and non-melanoma skin cancer: causal agents or innocent bystanders?. Front. Microbiol..

[16] Bouwes Bavinck JN (2018). Human papillomavirus and posttransplantation cutaneous squamous cell carcinoma: a multicenter, prospective cohort study. Am. J. Transpl..

[17] Hartevelt MM, Bavinck JN, Kootte AM, Vermeer BJ, Vandenbroucke JP (1990). Incidence of skin cancer after renal transplantation in The Netherlands. Transplantation.

[18] Tessari G (2010). Incidence and clinical predictors of a subsequent nonmelanoma skin cancer in solid organ transplant recipients with a first nonmelanoma skin cancer: a multicenter cohort study. Arch. Dermatol..

[19] Lanz J (2019). Aggressive squamous cell carcinoma in organ transplant recipients. JAMA Dermatol..

[20] Blessing K (1989). Histopathology of skin lesions in renal allograft recipients-an assessment of viral features and dysplasia. Histopathology.

[21] Lowe SM (2012). Acquired epidermodysplasia verruciformis due to multiple and unusual HPV infection among vertically-infected, HIV-positive adolescents in Zimbabwe. Clin. Infect. Dis..

[22] de Jong SJ (2018). Epidermodysplasia verruciformis: inborn errors of immunity to human beta-papillomaviruses. Front. Microbiol..

[23] Arnold AW, Hofbauer GF (2012). Human papillomavirus and squamous cell cancer of the skin-epidermodysplasia verruciformis-associated human papillomavirus revisited. Curr. Probl. Dermatol..

[24] Harwood CA (2000). Human papillomavirus infection and non-melanoma skin cancer in immunosuppressed and immunocompetent individuals. J. Med. Virol..

[25] Bouwes Bavinck JN (2010). Multicenter study of the association between betapapillomavirus infection and cutaneous squamous cell carcinoma. Cancer Res..

[26] Karagas MR (2010). Genus beta human papillomaviruses and incidence of basal cell and squamous cell carcinomas of skin: population based case-control study. BMJ (Clin. Res. ed.).

[27] Waterboer T (2008). Serological association of beta and gamma human papillomaviruses with squamous cell carcinoma of the skin. Br. J. Dermatol..

[28] Horev L (2015). Generalized verrucosis and HPV-3 susceptibility associated with CD4 T-cell lymphopenia caused by inherited human interleukin-7 deficiency. J. Am. Acad. Dermatol..

[29] Alisjahbana B (2010). Disfiguring generalized verrucosis in an indonesian man with idiopathic CD4 lymphopenia. Arch. Dermatol..

[30] WHO-ONT. 2019 Global Observatory on Donation and Transplantation (2021). http://www.transplant-observatory.org/wp-content/uploads/2021/06/GODT2019-data_web_updated-June-2021.pdf.

[31] Scott Bentley, T. & Hanson, S. G. 2011 U.S. organ and tissue transplant cost estimates and discussion. (Milliman, 2011).

[32] DSO. Jahresbericht: Organspende und Transplantation in Deutschland 2021 (2022).

[33] Kasiske BL, Cohen D, Lucey MR, Neylan JF (2000). Payment for immunosuppression after organ transplantation. American Society of Transplantation. Jama.

[34] Ramsay HM, Fryer AA, Hawley CM, Smith AG, Harden PN (2002). Non-melanoma skin cancer risk in the Queensland renal transplant population. Br. J. Dermatol..

[35] Harwood CA (2013). A surveillance model for skin cancer in organ transplant recipients: a 22-year prospective study in an ethnically diverse population. Am. J. Transpl..

[36] Mudigonda T, Pearce DJ, Yentzer BA, Williford P, Feldman SR (2010). The economic impact of non-melanoma skin cancer: a review. J. Natl Compr. Cancer Netw..

[37] Rubio I (2011). The N-terminal region of the human papillomavirus L2 protein contains overlapping binding sites for neutralizing, cross-neutralizing and non-neutralizing antibodies. Virology.

[38] Pouyanfard, S. et al. Minor capsid protein L2 polytope induces broad protection against oncogenic and mucosal human papillomaviruses. *J. Virol.* **92**, 10.1128/jvi.01930-17 (2018). 10.1128/JVI.01930-17 PMC5790957 29212932

[39] Zhao X (2020). Combined prophylactic and therapeutic immune responses against human papillomaviruses induced by a thioredoxin-based L2-E7 nanoparticle vaccine. PLoS Pathog..

[40] Spagnoli G (2017). Broadly neutralizing antiviral responses induced by a single-molecule HPV vaccine based on thermostable thioredoxin-L2 multiepitope nanoparticles. Sci. Rep..

[41] Gambhira R (2007). A protective and broadly cross-neutralizing epitope of human papillomavirus L2. J. Virol..

[42] Huber B (2017). Chimeric L2-based virus-like particle (VLP) vaccines targeting cutaneous human papillomaviruses (HPV). PLoS ONE.

[43] Seitz H (2014). A three component mix of thioredoxin-L2 antigens elicits broadly neutralizing responses against oncogenic human papillomaviruses. Vaccine.

[44] Rubio I (2009). Potent anti-HPV immune responses induced by tandem repeats of the HPV16 L2 (20–38) peptide displayed on bacterial thioredoxin. Vaccine.

[45] Wang JW (2015). Production of furin-cleaved papillomavirus pseudovirions and their use for in vitro neutralization assays of L1- or L2-specific antibodies. Curr. Protoc. Microbiol..

[46] Wang, J. W. et al. Roles of Fc domain and exudation in L2 antibody-mediated protection against human papillomavirus. *J. Virol.* **92**, 10.1128/jvi.00572-18 (2018). 10.1128/JVI.00572-18 PMC6052307 29743371

[47] Pfister H, zur Hausen H (1978). Seroepidemiological studies of human papilloma virus (HPV-1) infections. Int. J. Cancer.

[48] Lin YL, Borenstein LA, Selvakumar R, Ahmed R, Wettstein FO (1992). Effective vaccination against papilloma development by immunization with L1 or L2 structural protein of cottontail rabbit papillomavirus. Virology.

[49] Campo MS (1997). Vaccination against papillomavirus in cattle. Clin. Dermatol..

[50] Harnacker J, Hainisch EK, Shafti-Keramat S, Kirnbauer R, Brandt S (2017). Type-specific L1 virus-like particle-mediated protection of horses from experimental bovine papillomavirus 1-induced pseudo-sarcoid formation is long-lasting. J. Gen. Virol..

[51] Chahoud J (2016). Association between β-genus human papillomavirus and cutaneous squamous cell carcinoma in immunocompetent individuals-A meta-analysis. JAMA Dermatol..

[52] Andersson K (2012). Prospective study of human papillomavirus seropositivity and risk of nonmelanoma skin cancer. Am. J. Epidemiol..

[53] Farzan SF (2013). Cutaneous alpha, beta and gamma human papillomaviruses in relation to squamous cell carcinoma of the skin: a population-based study. Int. J. Cancer.

[54] Rollison, D. E. et al. Cutaneous human papillomaviruses and the risk of keratinocyte carcinomas. *Cancer Res.*10.1158/0008-5472.Can-21-0805 (2021). 10.1158/0008-5472.CAN-21-0805 PMC8416805 34266893

[55] Proby CM (2011). A case-control study of betapapillomavirus infection and cutaneous squamous cell carcinoma in organ transplant recipients. Am. J. Transpl..

[56] Genders RE (2015). The presence of betapapillomavirus antibodies around transplantation predicts the development of keratinocyte carcinoma in organ transplant recipients: a cohort study. J. investigative Dermatol..

[57] Vinzón SE (2014). Protective vaccination against papillomavirus-induced skin tumors under immunocompetent and immunosuppressive conditions: a preclinical study using a natural outbred animal model. PLoS Pathog..

[58] Ghittoni R, Accardi R, Chiocca S, Tommasino M (2015). Role of human papillomaviruses in carcinogenesis. Ecancermedicalscience.

[59] Strickley JD (2019). Immunity to commensal papillomaviruses protects against skin cancer. Nature.

[60] Yang F (2020). Broad neutralization responses against oncogenic human papillomaviruses induced by a minor capsid L2 polytope genetically incorporated into bacterial ferritin nanoparticles. Front. Immunol..

[61] Lunn A, Ravenscroft J, Watson AR (2010). Cutaneous warts in children before and after renal transplantation. Pediatr. Nephrol..

[62] Michael KM (2008). Seroprevalence of 34 human papillomavirus types in the German general population. PLoS Pathog..

[63] Danziger-Isakov L, Kumar D (2019). Vaccination of solid organ transplant candidates and recipients: Guidelines from the American society of transplantation infectious diseases community of practice. Clin. Transplant..

[64] Hofmeyer T (2013). Arranged sevenfold: structural insights into the C-terminal oligomerization domain of human C4b-binding protein. J. Mol. Biol..

[65] Ogun SA, Dumon-Seignovert L, Marchand JB, Holder AA, Hill F (2008). The oligomerization domain of C4-binding protein (C4bp) acts as an adjuvant, and the fusion protein comprised of the 19-kilodalton merozoite surface protein 1 fused with the murine C4bp domain protects mice against malaria. Infect. Immun..

[66] Liu S (1997). Removal of endotoxin from recombinant protein preparations. Clin. Biochem..

[67] Buck CB (2006). Carrageenan is a potent inhibitor of papillomavirus infection. PLoS Pathog..

[68] Sehr P (2013). High-throughput pseudovirion-based neutralization assay for analysis of natural and vaccine-induced antibodies against human papillomaviruses. PloS ONE.

