# Supplementary material for: A broadly protective vaccine against cutaneous human papillomaviruses
Source: NPJ Vaccines. 2022 Oct 10;7:116. doi: 10.1038/s41541-022-00539-0 (PMC9550855; doi:10.1038/s41541-022-00539-0)
Supplement: Supplementary file 1 — Supplementary Material [file 41541_2022_539_MOESM1_ESM.pdf]

**Supplementary information**

# A Broadly Protective Vaccine against Cutaneous Human Papillomaviruses

Filipe Colaco Mariz[1]*, Kathrin Balz[1], Manuela Dittrich[1], Yueru Zhang[1], Fan Yang[1], Xueer Zhao[1], Angelo Bolchi[2], Simone Ottonello[2], Martin Müller[1]*

***Correspondence**: Martin Müller and Filipe Colaco Mariz, Tumorvirus-specific Vaccination Strategies, Deutsches Krebsforschungszentrum, Im Neuenheimer Feld 280, 69120 Heidelberg, Germany. E-mail: martin.mueller@dkfz.de and f.mariz@dkfz.de. Telephone: +49 6221 42-4628.

This PDF includes:

Supplementary Figures 1 to 4

Supplementary Tables 1 to 7

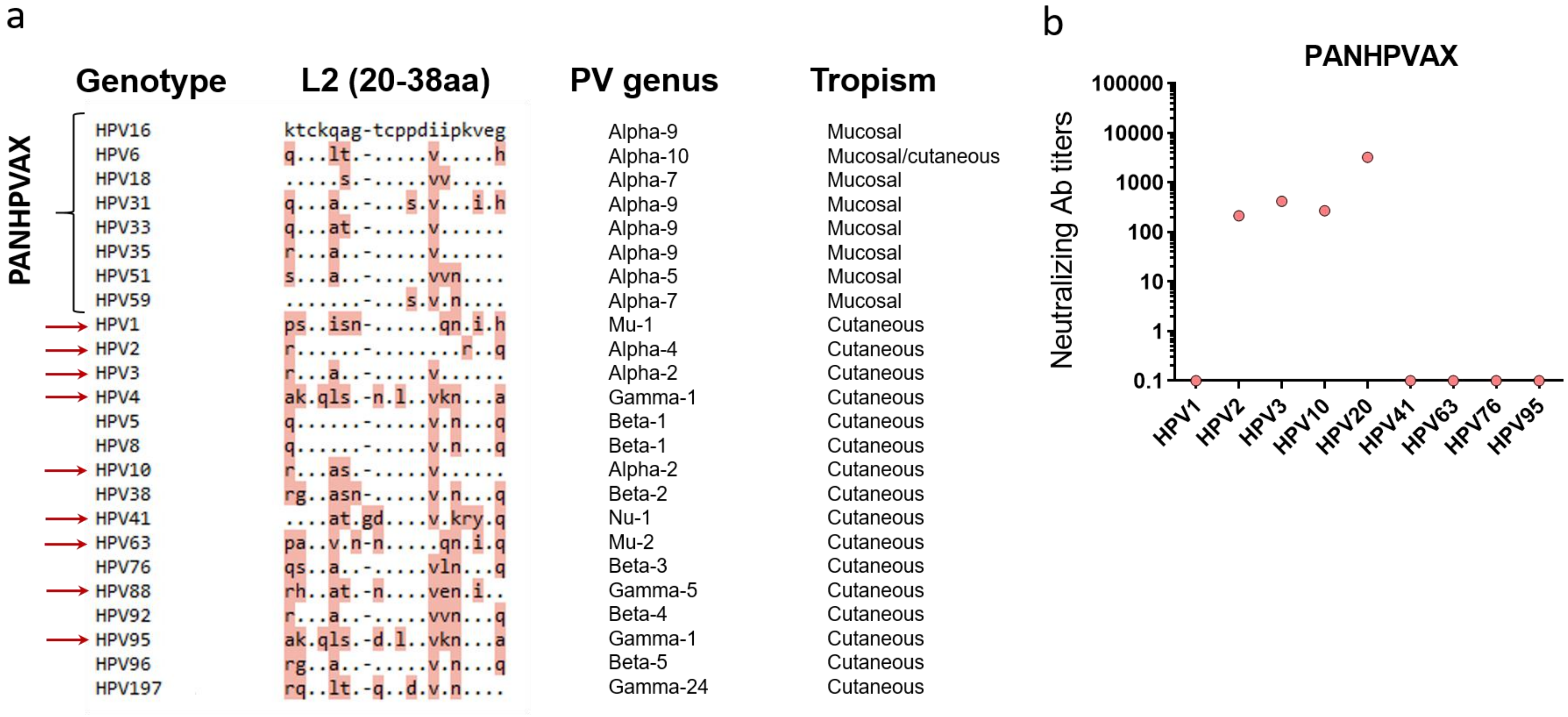


**Supplementary Figure 1. Amino acid variations within the L2 aa 20-38 region correlate with the limited cross-neutralizing immune responses against cutaneous HPV types induced by the PANHPVAX vaccine. (a)** Alignment of of the L2 aa 20-38 regions of the eight mucosal HPV (alpha genus) L2 epitopes comprised within the PANHPVAX antigen and of 16 cutaneous HPV types belonging to different genera. The HPV types targeted by the two initial candidate vaccines (PfTrx-L2c6mer and PfTrx-L2c9mer) are marked with red arrows. **(b)** Cross-neutralizing antibody titers to nine cutaneous HPV types measured by the L1-PBNA in a pool of sera derived from 10 Balb/c mice immunized with the PANHPVAX vaccine.

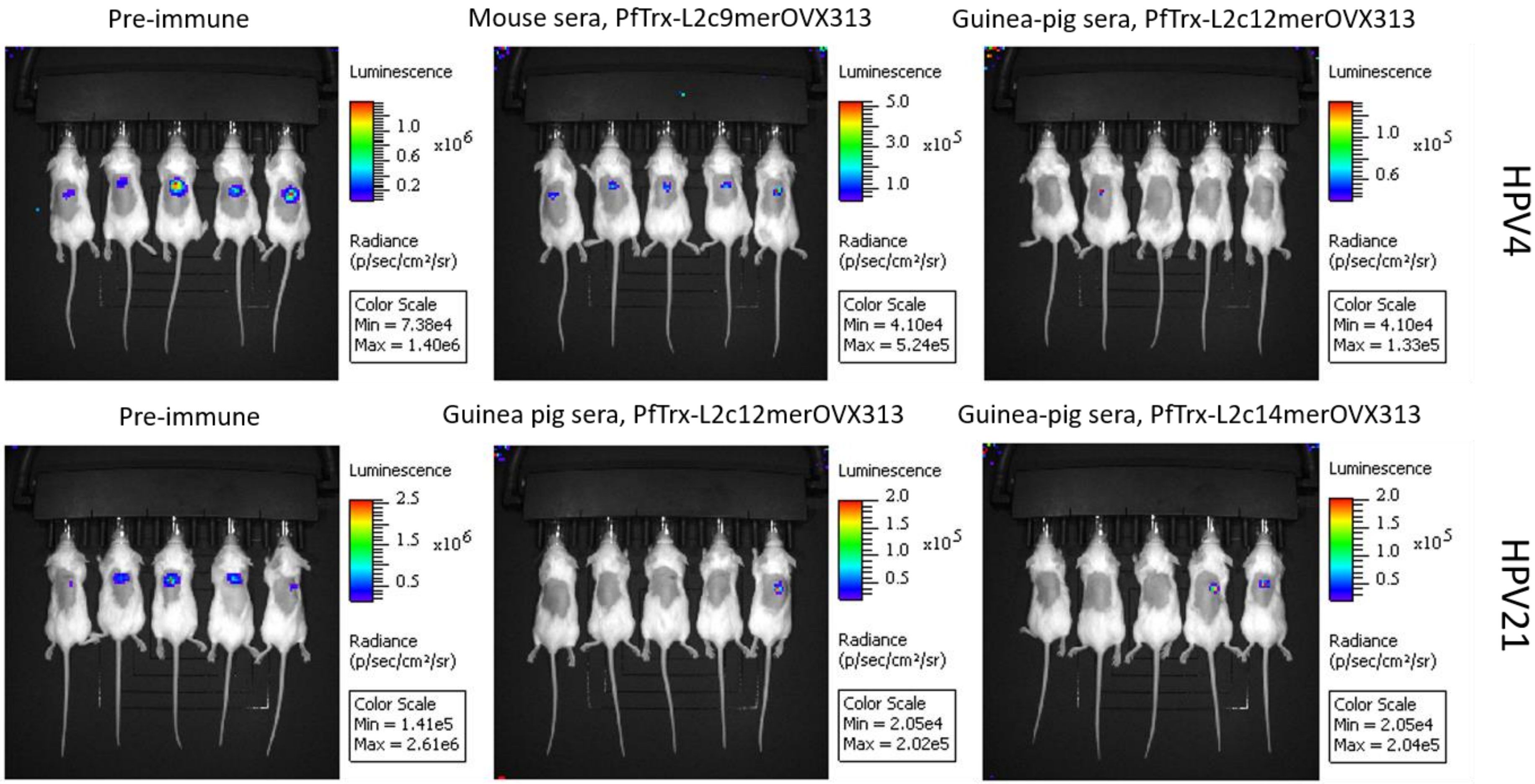

Pre-immune
Mouse sera, PfTrx-L2c9merOVX313
Guinea-pig sera, PfTrx-L2c12merOVX313
HPV4
Luminescence
1.0
0.6
0.2
x10^6
Radiance (p/sec/cm²/sr)
Color Scale Min = 7.38e4 Max = 1.40e6
5.0
3.0
1.0
x10^5
Color Scale Min = 4.10e4 Max = 5.24e5
0.6
Color Scale Min = 4.10e4 Max = 1.33e5
Pre-immune
Guinea pig sera, PfTrx-L2c12merOVX313
Guinea-pig sera, PfTrx-L2c14merOVX313
HPV21
2.5
1.5
0.5
Color Scale Min = 1.41e5 Max = 2.61e6
2.0
Color Scale Min = 2.05e4 Max = 2.02e5
Color Scale Min = 2.05e4 Max = 2.04e5

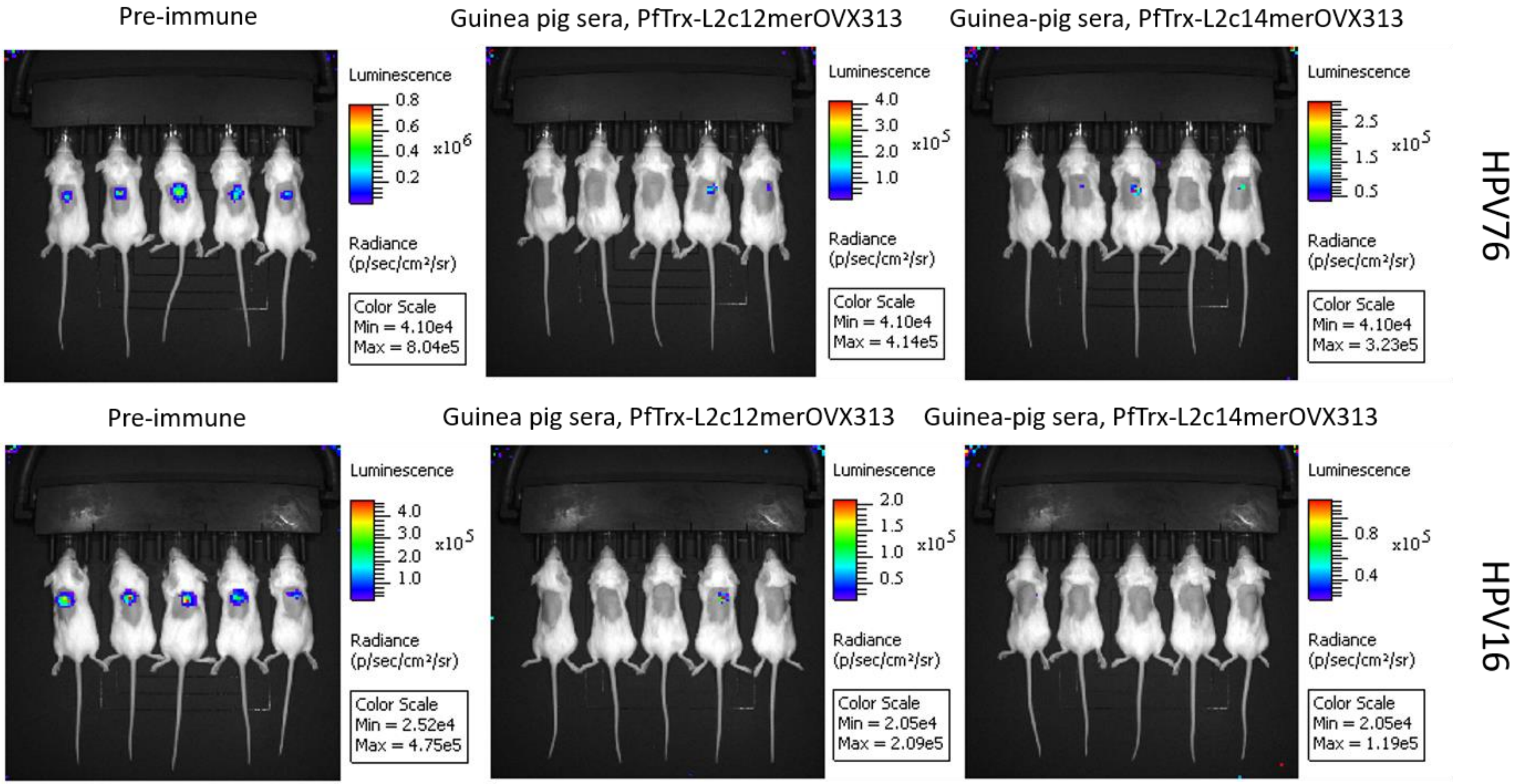


**Supplementary Figure 2. *In vivo* protection against cutaneous challenge.** Naïve mice were passively immunized with pools of sera derived from either 10 mice or 2 guinea pigs immunized with the PfTrx-L2c9merOVX313, PfTrx-L2c12merOVX313 or PfTrx-L2c14merOVX313 antigen as indicated. Protection against cutaneous challenge with the indicated PSVs afforded by the immune sera is compared with that provided by sera from non-immunized mice (*pre-immune sera*). Colours (scale shown on the right) represent the intensity of luciferase expression upon transduction and challenge with the indicated PSVs as average radiance (photons per second per square centimeter per steradian).

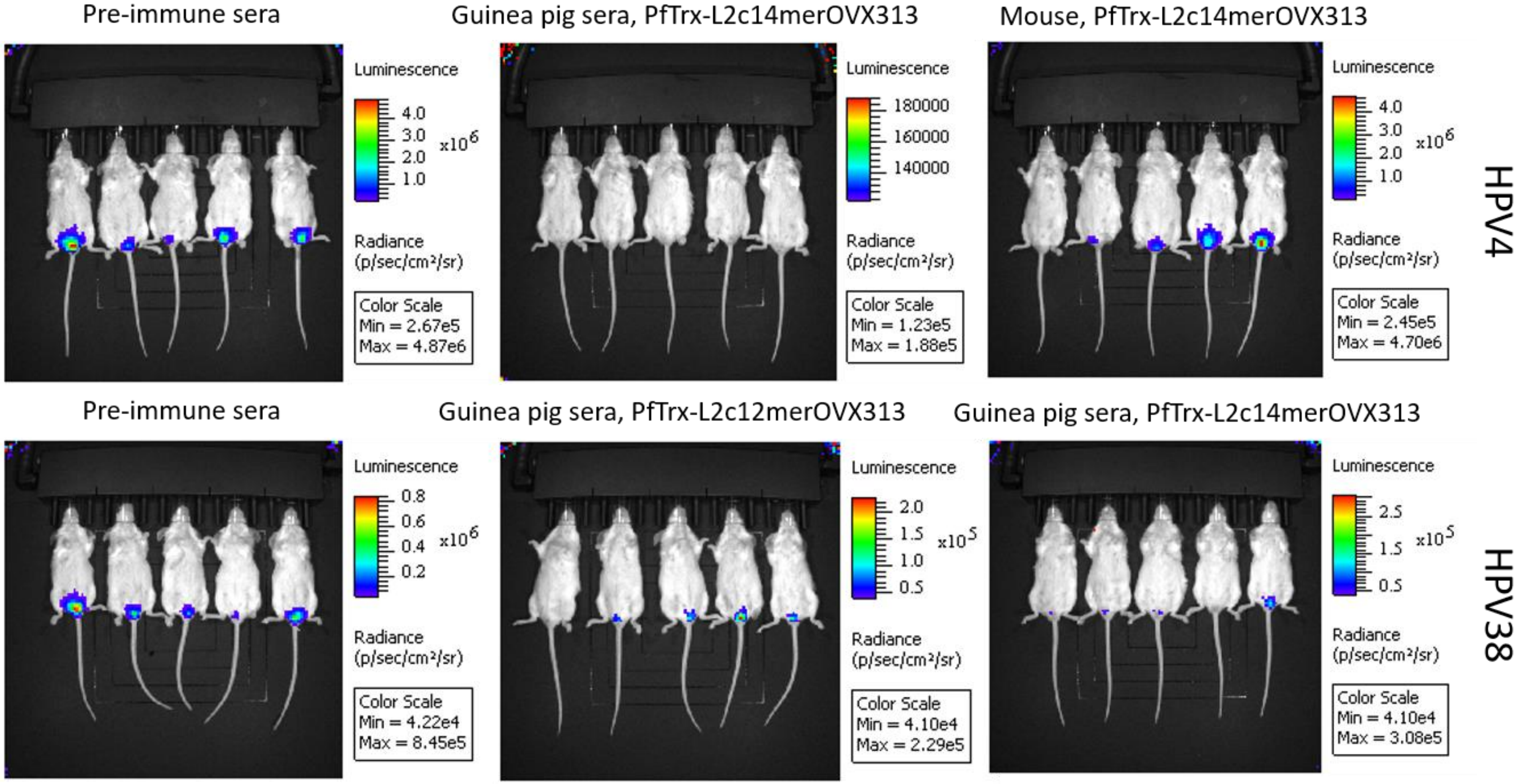


**Supplementary Figure 3. *In vivo* protection against intra-vaginal mouse challenge afforded by sera from animals immunized with the two high-complexity antigens.** Naïve mice were passively immunized with pools of sera derived from either 10 mice or 2 guinea pigs immunized with the PfTrx-L2c12merOVX313 or the PfTrx-L2c14merOVX313 antigen as indicated. Protection against cervico-vaginal challenge with HPV4 and HPV38 PSV types afforded by the immune sera is compared with that provided by sera from non-immunized mice (*pre-immune sera*). Colors (scale shown on the right) represent the intensity of luciferase expression upon transduction and challenge with the indicated PSVs as average radiance (photons per second per square centimeter per steradian).

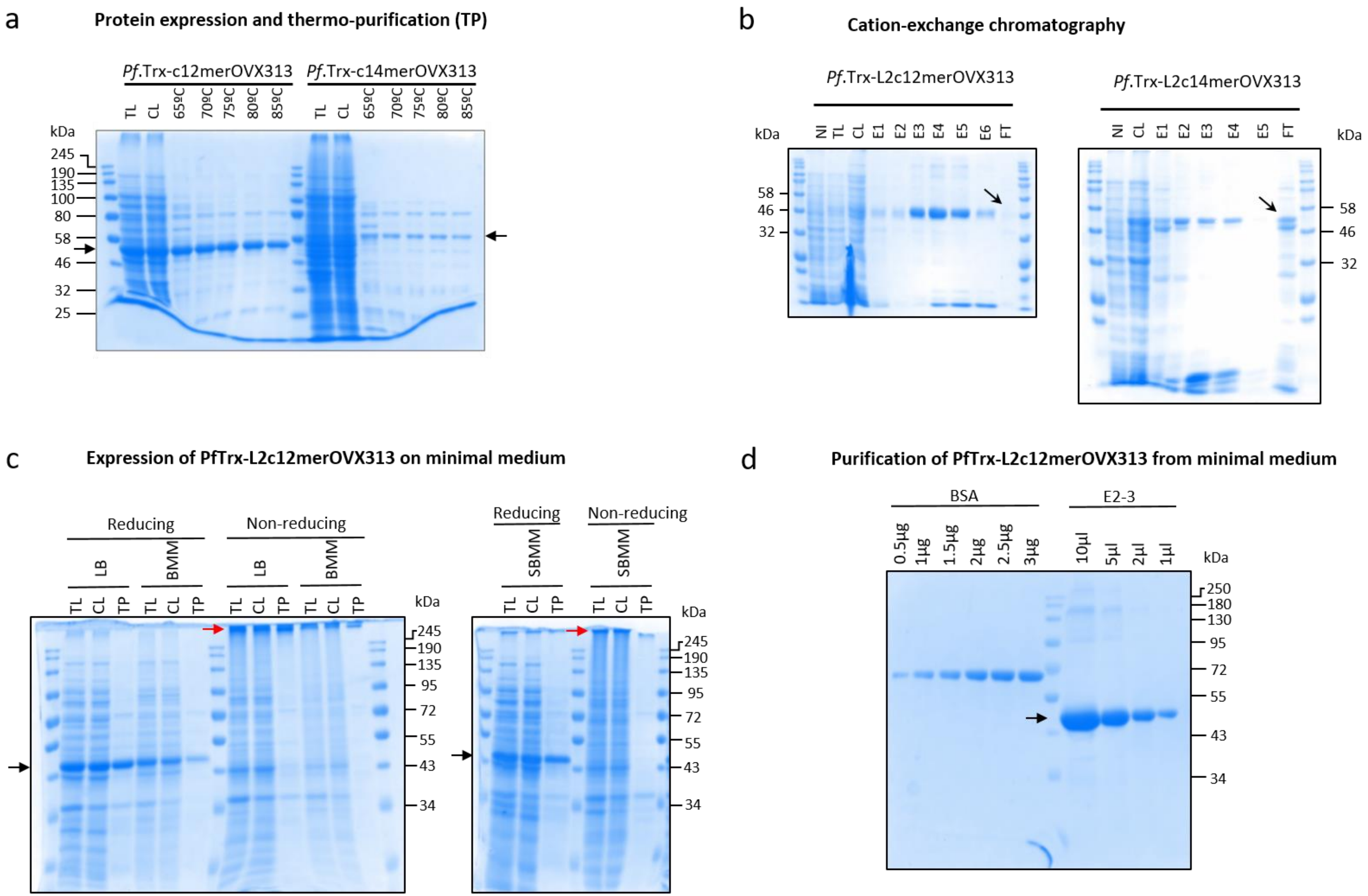


**Supplementary Figure 4. GMP-compatible production properties of the PfTrx-L2c12merOVX313 candidate vaccine. (a)** Total lysate (TL) and cleared lysate (CL; i.e., soluble fraction) from IPTG-induced bacteria expressing the 12mer or 14mer antigens as indicated. Also shown are the partially purified products obtained

by thermal-purification (TP) at different temperatures. **(b)** Peak-eluting (E1-6; balanced protein inputs measured by Bradford colorimetric quantification) and flow-through (FT) fractions obtained from cation-exchange chromatography of the thermally purified PfTrx-L2c12merOVX313 and PfTrx-L2c14merOVX313 products, analyzed side-by-side with aliquots of TL and CL. Black arrows point to the presence or absence of the target proteins in the FT fraction. (**c**) SDS-PAGE analysis under reducing or non-reducing conditions of the indicated fractions (TL, CL and TP-80°C) of the PfTrx-L2c12merOVX313 antigen extracted from bacteria cultured in standard LB, in proprietary minimal BMM or in soya-supplemented BMM (SBMM) media; black and red arrows, respectively, indicate the monomeric and the oligomeric forms of the antigen. (**d**) Different amounts of the pooled peak-eluting fractions (E2-3) were analyzed by SDS-PAGE (right-side lanes) and quantified by comparison with known amounts of bovine serum albumin (BSA; left-side lanes); black arrow indicates the PfTrx-L2c12merOVX313 protein band.

**Supplementary Table 1**. Levels of neutralizing antibodies against 10 cutaneous HPV types elicited in mice by the four candidate vaccines measured by the L1-PBNA[1].

| Antigens | Mean neutralizing Ab titers (standard error of mean, SEM) | | | | | | | | | |
|---|---|---|---|---|---|---|---|---|---|---|
| | **HPV2** | **HPV3** | **HPV4** | **HPV5** | **HPV20** | **HPV41** | **HPV63** | **HPV92** | **HPV95** | **HPV96** |
| **PfTrx-L2c6mer** | - | - | - | 130 (130) | 670 (650) | - | - | 330 (240) | - | 100 (100) |
| **PfTrx-L2c9mer** | - | - | - | 110 (70) | - | - | - | 90 (40) | - | - |
| **PfTrx-L2c6merOVX313** | 150 (40) | 90 (20) | - | 850 (260) | 510 ( 170) | - | - | 630 (190) | - | 710 (560) |
| **PfTrx-L2c9merOVX313** | 130 (30) | 80 (40) | - | 670 (130) | 1570 (900) | 1160 (590) | 530 (370) | 1010 (220) | 80 (50) | 140 (60) |

[1]Mean antibody titers and corresponding standard error of the mean (SEM) for each HPV type were calculated for groups of 10 immune-sera.

**Supplementary Table 2**. Percentage (%) of responders and corresponding 95% confidence intervals (CI) to 10 cutaneous HPV types measured by the L1-PBNA on sera from 10 mice immunized with the four different candidate vaccines.

| Antigens | % of responders (95% CI) | | | | | | | | | |
|---|---|---|---|---|---|---|---|---|---|---|
| | **HPV2** | **HPV3** | **HPV4** | **HPV5** | **HPV20** | **HPV41** | **HPV63** | **HPV92** | **HPV95** | **HPV96** |
| **PfTrx-L2c6mer** | 10 (0-44) | 20 (2-55) | 0 (0-30) | 10 (0-44) | 20 (2-55) | 0 (0-30) | 10 (0-44) | 50 (18-81) | 0 (0-30) | 10 (0-44) |
| **PfTrx-L2c9mer** | 10 (0-44) | 0 (0-30) | 0 (0-30) | 30 (6-65) | 0 (0-30) | 0 (0-30) | 20 (2-55) | 50 (18-81) | 20 (2-55) | 0 (0-30) |
| **PfTrx-L2c6merOVX313** | 70 (34-93) | 80 (44-97) | 0 (0-30) | 100 (70-100) | 70 (34-93) | 0 (0-30) | 40 (12-73) | 100 (70-100) | 50 (18-81) | 70 (34-93) |
| **PfTrx-L2c9merOVX313** | 80 (44-97) | 90 (55-100) | 0 (0-30) | 90 (55-100) | 70 (34-93) | 80 (44-97) | 70 (34-93) | 100 (70-100) | 40 (12-73) | 80 (44-97) |

**Supplementary Table 3**. Levels of neutralizing antibodies against 13 cutaneous HPV types elicited in guinea pigs by the four candidate vaccines measured by the L1-PBNA[1]

| Antigens | Mean neutralizing Ab titers (standard error of mean, SEM) | | | | | | | | | | | | |
|---|---|---|---|---|---|---|---|---|---|---|---|---|---|
| | HPV1 | HPV2 | HPV3 | HPV4 | HPV5 | HPV20 | HPV38 | HPV41 | HPV63 | HPV76 | HPV92 | HPV95 | HPV96 |
| **PfTrx-L2c6mer** | 290 (290) | 1376 (520) | 1259 (651) | - | 9243 (3503) | 12000 (0) | - | - | 1728 (246) | - | - | - | 4850 (2837) |
| **PfTrx-L2c9mer** | 231 (167) | 1051 (467) | 560 (256) | - | 2098 (2098) | 9438 (2561) | - | 298 (207) | 8030 (3970) | - | 6000 (6000) | 66 (66) | 1730 (1367) |
| **PfTrx-L2c6merOVX313** | 71 (21) | 901 (217) | 560 (256) | - | 1945 (1945) | 10799 (1200) | - | - | 4757 (4620) | - | 2475 (1431) | 87 (87) | 1043 (339) |
| **PfTrx-L2c9merOVX313** | 89 (2) | 2448 (85) | 2881 (512) | 219 (140) | 6000 (6000) | 12000 (0) | - | 1115 (675) | 8288 (3712) | - | 9719 (525) | 383 (39) | 8917 (2340) |

[1]Mean antibody titers and corresponding standard error of the mean (SEM) for each HPV type were calculated for groups of two immune-sera.

**Supplementary Table 4**. Levels of neutralizing antibodies against nine cutaneous HPV types elicited in mice by three different candidate vaccines measured by the L1-PBNA[1].

| Antigens | Mean neutralizing Ab titers (standard error of mean, SEM) | | | | | | | | |
|---|---|---|---|---|---|---|---|---|---|
| | **HPV2** | **HPV3** | **HPV4** | **HPV5** | **HPV41** | **HPV63** | **HPV92** | **HPV95** | **HPV96** |
| **PANHPVAX** | 150 (70) | 100 (40) | - | 710 (450) | - | - | 2300 (1360) | - | 610 (330) |
| **PfTrx-L2c6merOVX313** | 150 (40) | 90 (20) | - | 850 (260) | - | - | 630 (190) | - | 710 (560) |
| **PfTrx-L2c9merOVX313** | 130 (30) | 80 (10) | - | 670 (130) | 1160 (590) | 530 (370) | 1010 (220) | 80 (50) | 140 (60) |

[1]Mean antibody titers and corresponding standard error of the mean (SEM) for each HPV type were calculated for groups of 10 sera.

**Supplementary Table 5**. Percentage (%) of responders and corresponding 95% confidence intervals (CI) to nine cutaneous HPV types measured by the L1-PBNA on sera from 10 mice immunized with three different candidate vaccines.

| Antigens | % of responders (95% confidence interval) | | | | | | | | |
|---|---|---|---|---|---|---|---|---|---|
| | **HPV2** | **HPV3** | **HPV4** | **HPV5** | **HPV41** | **HPV63** | **HPV92** | **HPV95** | **HPV96** |
| **PANHPVAX** | 40 (12-73) | 50 (18-81) | 0 (0-30) | 50 (18-81) | 0 (0-30) | 10 (0-44) | 50 (18-81) | 0 (0-30) | 70 (34-93) |
| **PfTrx-L2c6merOVX313** | 70 (34-93) | 80 (44-97) | 0 (0-30) | 100 (70-100) | 0 (0-30) | 40 (12-73) | 100 (70-100) | 40 (12-73) | 70 (34-93) |
| **PfTrx-L2c9merOVX313** | 80 (44-97) | 90 (55-100) | 0 (0-30) | 90 (55-100) | 80 (44-97) | 70 (34-93) | 100 (70-100) | 40 (12-73) | 80 (44-97) |

**Supplementary Table 6**. Levels of neutralizing antibodies against 11 cutaneous HPV types elicited in mice by five different candidate vaccines measured by the L1-PBNA[1].

| **Antigens** | **Mean neutralizing Ab titers (standard error of mean, SEM)** | | | | | | | | | | |
|---|---|---|---|---|---|---|---|---|---|---|---|
| | **HPV2** | **HPV4** | **HPV9** | **HPV14** | **HPV20** | **HPV21** | **HPV36** | **HPV41** | **HPV63** | **HPV92** | **HPV124** |
| **PfTrx-L2c7merOVX313** | - | - | - | 1290 (1190) | 80 (30) | - | 60 (30) | 500 (270) | - | 720 (320) | - |
| **PfTrx-L2c9merOVX313** | - | - | - | 300 (80) | 230 (80) | - | 120 (40) | 1200 (530) | 720 (250) | 1650 (460) | - |
| **PfTrx-L2c10merOVX313** | 90 (40) | - | 70 (40) | 590 (185) | 670 (270) | 70 (40) | 450 (170) | 480 (230) | 50 (50) | 4100 (1260) | 70 (30) |
| **PfTrx-L2c12merOVX313** | - | - | 110 (30) | 520 (190) | 950 (420) | 60 (20) | 400 (230) | 230 (80) | - | 4430 (1430) | 100 (40) |
| **PfTrx-L2c14merOVX313** | 240 (140) | - | - | 1050 (250) | 660 (140) | 80 (20) | 600 (110) | 60 (20) | 100 (20) | 4170 (1090) | 70 (20) |

[1]Mean antibody titers and corresponding standard error of the mean (SEM) for each HPV type were calculated for groups of 10 sera (or less, in the case of HPV36, see Figure 4).

**Supplementary Table 7.** List of cutaneous HPV PSVs used to assess the immunogenicity of candidate vaccines by neutralization and murine challenge assays.

| Type | Activity/Infectivity[1] | | | Expression of capsid proteins[2] | |
|---|---|---|---|---|---|
| | PBNA | *In vivo* | FC-PBNA | L1 | L2 |
| HPV1 | - | NA | NA | + (4543/MD2H11) | + (162) |
| hHPV1[3] | + | NA | NA | NA | NA |
| HPV2 | - | NA | NA | + (4543/MD2H11) | + (162) |
| hHPV2[3] | + | NA | NA | NA | NA |
| HPV3 | + | NA | NA | + (4543/MD2H11) | + (K18/162) |
| HPV4 | + | + | + | + (4543/MD2H11) | + (162) |
| HPV5 | + | + | + | NA | NA |
| HPV8 | - | NA | NA | NC (MD2H11) | NC (162) |
| HPV9 | + | NA | NA | + (MD2H11) | + |
| HPV10 | + | NA | NA | + (4543/MD2H11) | + (162) |
| HPV14 | + | NA | NA | + (MD2H11) | + |
| HPV15 | - | NA | NA | + (MD2H11) | + (162) |
| HPV20 | + | NA | NA | NA | NA |
| HPV21 | + | + | NA | + (MD2H11) | + (162) |
| HPV22 | - | NA | NA | + (MD2H11) | + |
| HPV27 | - | NA | NA | NA | NA |
| HPV36 | + | NA | NA | + (MD2H11) | + (162) |
| HPV38 | + | + | + | NA | NA |
| HPV41 | - | NA | NA | + (MD2H11) | + (162) |
| hHPV41[3] | + | NA | NA | NA | NA |
| HPV48 | + | NA | + | + (4543/MD2H11) | NC |
| HPV63 | + | NA | NA | + (MD2H11/4543) | + (162) |
| HPV75 | + | NA | + | + (MD2H11) | + (162) |
| HPV76 | + | + | + | NA | NA |

| | | | | | |
|---|---|---|---|---|---|
| HPV88 | - | NA | NA | - (MD2H11) | + (K18/162) |
| hHPV88[3] | - | NA | NA | NA | NA |
| HPV92 | + | NA | NA | NA | NA |
| HPV93 | - | NA | NA | - (MD2H11) | - (162) |
| HPV95 | + | NA | + | + (4543/MD2H11) | + (162) |
| HPV96 | + | NA | + | NA | NA |
| HPV115 | - | NA | NA | - (MD2H11) | + (162) |
| HPV124 | + | NA | NA | + (MD2H11) | + (162) |
| HPV172 | + | NA | NA | - (MD2H11) | + (162) |
| HPV197 | + | NA | NA | + (MD2H11) | - (162) |

[1]Infective and non-infective PSVs are indicated as + or -, respectively; NA: not-assessed; NC: not-conclusive result.

[2]Expression of capsid proteins was assessed by immuno-blotting performed on the lysates of 293TT cells transfected with the respective PSV constructs using a set of anti-L1 and anti-L2 monoclonal and polyclonal antibodies.

[3]"Hybrid" PSV constructs were designed, in which HPV3 L2 (aa 1-46) was replaced by the corresponding L2 regions of HPV1, HPV2 and HPV41. This strategy was also applied to hHPV88 but did not yield infective PSVs.
