# Supplementary material for: A broadly protective vaccine against cutaneous human papillomaviruses
Source: NPJ Vaccines. 2022 Oct 10;7:116. doi: 10.1038/s41541-022-00539-0 (PMC9550855; doi:10.1038/s41541-022-00539-0)
Supplement: Supplementary file 2 — REPORTING SUMMARY [file 41541_2022_539_MOESM2_ESM.pdf]

nature portfolio

Corresponding author(s): Mariz FC, Müller M: NPJVACCINES-02072R

Last updated by author(s): Aug 21, 2022

Reporting Summary

Nature Portfolio wishes to improve the reproducibility of the work that we publish. This form provides structure for consistency and transparency in reporting. For further information on Nature Portfolio policies, see our Editorial Policies and the Editorial Policy Checklist.

Statistics

For all statistical analyses, confirm that the following items are present in the figure legend, table legend, main text, or Methods section.

n/a	Confirmed	
☐	☒	The exact sample size (*n*) for each experimental group/condition, given as a discrete number and unit of measurement
☐	☒	A statement on whether measurements were taken from distinct samples or whether the same sample was measured repeatedly
☐	☒	The statistical test(s) used AND whether they are one- or two-sided<br>*Only common tests should be described solely by name; describe more complex techniques in the Methods section.*
☒	☐	A description of all covariates tested
☒	☐	A description of any assumptions or corrections, such as tests of normality and adjustment for multiple comparisons
☐	☒	A full description of the statistical parameters including central tendency (e.g. means) or other basic estimates (e.g. regression coefficient) AND variation (e.g. standard deviation) or associated estimates of uncertainty (e.g. confidence intervals)
☐	☒	For null hypothesis testing, the test statistic (e.g. *F*, *t*, *r*) with confidence intervals, effect sizes, degrees of freedom and *P* value noted<br>*Give P values as exact values whenever suitable.*
☒	☐	For Bayesian analysis, information on the choice of priors and Markov chain Monte Carlo settings
☒	☐	For hierarchical and complex designs, identification of the appropriate level for tests and full reporting of outcomes
☒	☐	Estimates of effect sizes (e.g. Cohen's *d*, Pearson's *r*), indicating how they were calculated

Our web collection on statistics for biologists contains articles on many of the points above.

Software and code

Policy information about availability of computer code

Data collection	Living Image 2.50.1 software (Xenogen, 465 PerkinElmer); Image Lab 5.2.1 (Biorad); Unicorn 5.0 software (Amersham, GE Healthcare).
Data analysis	GraphPad Prism 8.3.1

For manuscripts utilizing custom algorithms or software that are central to the research but not yet described in published literature, software must be made available to editors and reviewers. We strongly encourage code deposition in a community repository (e.g. GitHub). See the Nature Portfolio guidelines for submitting code & software for further information.

Data

Policy information about availability of data

All manuscripts must include a data availability statement. This statement should provide the following information, where applicable:

- Accession codes, unique identifiers, or web links for publicly available datasets
- A description of any restrictions on data availability
- For clinical datasets or third party data, please ensure that the statement adheres to our policy

The authors confirm that the raw data of this study were generated at the DKFZ. In addition to the Supplementary Tables and Supplementary Figures, further data supporting the findings of this study are available upon reasonable request to the corresponding authors according to DKFZ data safety protection regulations.

Human research participants

Policy information about studies involving human research participants and Sex and Gender in Research.

Reporting on sex and gender	Not applicable.
Population characteristics	Not applicable.
Recruitment	Not applicable.
Ethics oversight	Not aplicable.

Note that full information on the approval of the study protocol must also be provided in the manuscript.

Field-specific reporting

Please select the one below that is the best fit for your research. If you are not sure, read the appropriate sections before making your selection.

☒ Life sciences ☐ Behavioural & social sciences ☐ Ecological, evolutionary & environmental sciences

For a reference copy of the document with all sections, see nature.com/documents/nr-reporting-summary-flat.pdf

Life sciences study design

All studies must disclose on these points even when the disclosure is negative.

Sample size	Sample size for each experiment is indicated in the material and methods and corresponding figure legends. No statistical methods were used to predetermine sample size, which was based on previous experience of the group.
Data exclusions	No data were excluded from the analysis.
Replication	All experiments were performed at least in duplicate and were successful.
Randomization	Animals subjected to experimentation were randomly divided into experimental groups.
Blinding	Investigators were not blinded to animal groups during experiments. The reported data for the animal experiments are not subjective but rather based on quantitative antigen/immunogen, neutralizing antibody titers and average luminescence radiance.

Reporting for specific materials, systems and methods

We require information from authors about some types of materials, experimental systems and methods used in many studies. Here, indicate whether each material, system or method listed is relevant to your study. If you are not sure if a list item applies to your research, read the appropriate section before selecting a response.

Materials & experimental systems

n/a	Involved in the study	
☐	☒	Antibodies
☐	☒	Eukaryotic cell lines
☒	☐	Palaeontology and archaeology
☐	☒	Animals and other organisms
☒	☐	Clinical data
☒	☐	Dual use research of concern

Methods

n/a	Involved in the study	
☒	☐	ChIP-seq
☒	☐	Flow cytometry
☒	☐	MRI-based neuroimaging

Antibodies

Antibodies used	Mouse monoclonal antibody generated in our laboratory: anti-HPV16 L2 antibody clone K18.
Validation	ELISA, PBNA (Rubio et al Virology 409, 348-359, doi:10.1016/j.virol.2010.10.017 (2011).

Eukaryotic cell lines

Policy information about cell lines and Sex and Gender in Research

Cell line source(s)	Cell lines generated in our laboratory:<br>1. HEK-293TT<br>2. HeLa TK4<br><br>Cell lines generated/obtained from others:<br>3. HEK-293TTF<br>4. LoVoT
Authentication	SNP-based human cell line authentication test was performed for cell lines generated in our lab. Cell lines generated/ obtained from others were not authenticated.
Mycoplasma contamination	Absence of cell culture contaminations of the cell line was determined by the Multiplex cell Contamination Test.
Commonly misidentified lines (See ICLAC register)	No commonly misidentified cell lines were used in this study.

Animals and other research organisms

Policy information about studies involving animals; ARRIVE guidelines recommended for reporting animal research, and Sex and Gender in Research

Laboratory animals	Six- to eight-weeks-old female BALB/c mice and 150-200g outbread Hartley (Crl:HA) female guinea pigs were purchased from Charles River Laboratories (Sulzfeld, Germany).
Wild animals	No wild animals were used in this study.
Reporting on sex	Only female animals were used in this study. The decision was based on previous experience/experiments of the group.
Field-collected samples	No field-collected sample was used in this study.
Ethics oversight	Experiments in mice and guinea pigs were approved by the Regierungspräsidium Karlsruhe (Germany) via animal permits G248/16 and A2/17, respectively. Animals were kept under specific-pathogen-free conditions, and all manipulations were strictly conducted in compliance with the animal ethics guidelines and approved protocols.

Note that full information on the approval of the study protocol must also be provided in the manuscript.
